# Generalization of Titanocene-Catalyzed Arylation of Epoxides through Radical Polar Crossover by Consecutive Paired Electrolysis

**DOI:** 10.1021/jacsau.6c00215

**Published:** 2026-06-11

**Authors:** Niklas Schmickler, Darryl F. Nater, Siegfried R. Waldvogel, Andreas Gansäuer

**Affiliations:** † Kekulé-Institut für Organische Chemie und Biochemie, Universität Bonn, Gerhard-Domagk-Straße 1, 53121 Bonn, Germany; ‡ Department of Electrosynthesis, Waldvogel Max-Planck-Institute for Chemical Energy Conversion, Stiftstraße 34−36, 45470 Mülheim an der Ruhr, Germany; § Institute of Biological and Chemical Systems − Functional Molecular Systems (IBCS-FMS), Karlsruhe Institute of Technology, Kaiserstraße 12, 76131 Karlsruhe, Germany

**Keywords:** galvanostatic electrolysis, radical σ-complex, cationic σ-complex, electro-organic synthesis

## Abstract

Here, we describe the merging of titanocene-catalyzed radical arylation with electro-organic synthesis that combines the advantages of both fields. The critical mechanistic issue of the titanocene-catalyzed radical arylation, the efficiency of rearomatization by a proton-coupled electron transfer, can be efficiently resolved via a radical polar crossover. This is achieved in a unique manner by consecutive paired galvanostatic electrolysis through a decoupling of the electron and proton transfer steps by spatially separating the critical redox processes, the anodic oxidation of the radical σ-complex to the cationic σ-complex, and the mild *in situ* preparation of the active catalyst by cathodic reduction of Cp_2_TiCl_2_.

## Graphical abstract

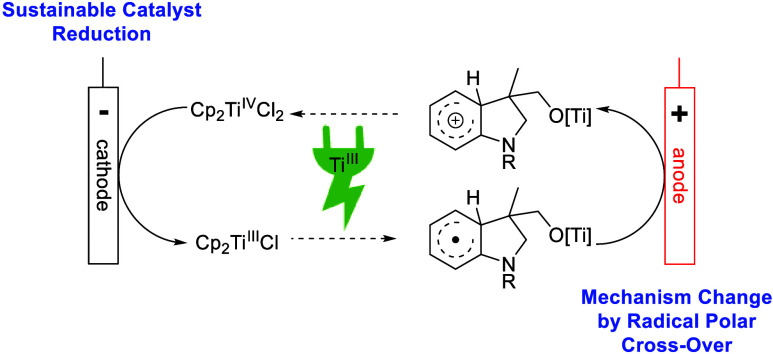

## 1 Introduction

The field of electro-organic synthesis is currently experiencing rapidly increasing interest in both industrial and academic laboratories. This is because it focuses on the direct use of electrical current in chemical reactions to induce and drive redox processes. Additionally, this approach shows highly attractive advantages, such as using sustainable energy sources, increasing process safety, and avoiding chemical waste.^1,2^ Transition-metal catalysis remains among the most powerful tools available to modern chemists, providing access to new and selective mechanistic pathways, including metal catalysis of radical reactions, and to vital classes of compounds. Combining the fields of synthetic electrochemistry with transition-metal catalysis is therefore ideally suited for implementing the sustainable and innovative approaches of electrochemistry to synthesis with control of reactivity and selectivity.^3^

In this respect, titanium complexes are particularly attractive because the catalysts show high stability in the electrochemical regime applied. Additionally, titanium is the second most abundant d-metal and essentially nontoxic.^4^ Moreover, its ligand sphere can be easily varied with a broad range of ligands to modify the steric and electronic properties of the complexes. Prominent classes of ligands are, for example, cyclopentadienyls or salens.^5^ Titanocene complexes in particular have been employed in electrochemical or chemical nitro reduction,^7^ dehalogenation,^8^ pinacol coupling,^7^ and epoxide opening reaction.^5b,6,9,10^

However, these applications only employ electrochemistry to generate the low-valent active species of the catalytic cycle at the cathode while ignoring the corresponding anodic process. This is partially due to the potentiostatic (constant voltage) setups employed in these cases that only provide control over the working electrode while offering no information about the counter electrode or the potential of the whole cell. However, when the electrolysis is performed under galvanostatic (constant current) conditions, the system is set to a certain current density and can adjust the potential of the whole cell to match the kinetics of the anodic and cathodic reactions, which directly provide information about the entire reaction system. Furthermore, this mode of operation is operationally simpler, with easily available devices, while also promising facile scalability.^11^ As such, we were interested to see whether we could develop a system that combines effective transition-metal catalysis with electrosynthesis while making use of both the anodic and cathodic reactions for controlling the catalysis.

The titanocene-catalyzed radical arylation of epoxides was chosen for this investigation as it is a particularly intriguing example of an atom-economical C–C bond formation (Scheme 1). It provides a convenient and very mild access to indolines and tetrahydroquinolines. Moreover, it has been demonstrated that both classes of heterocycles can be obtained enantiomerically pure and in high diastereomeric excesses through the merging of the arylation with regiodivergent epoxide opening.^12^

**Scheme 1:**
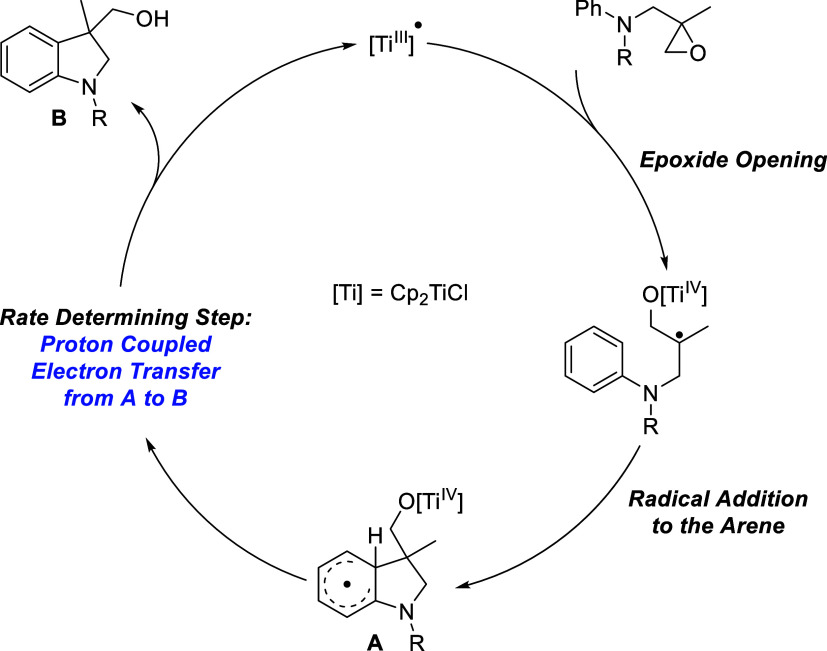
Catalytic Cycle of the Titanocene-Catalyzed Radical Arylation of Epoxides^12e^

This reaction is initiated by a reduction of the organic substrate by the titanocene­(III) catalyst, resulting in the Ti­(IV) complex. The catalyst is reduced back to Ti­(III) after radical translocation in the PCET step, leading to rearomatization. However, this rate-determining PCET step depends on the oxidizing power of the pending Ti­(IV) in **A**. Since titanocene­(IV) complexes are only weak oxidants in general, modifying the oxidation potential through ligands has clear limits.^13^ Therefore, the reaction is restricted to a handful of electron-rich indolines, which restrains their use in a multitude of pharmaceutically active compounds.^14^ An alternative approach to this problem is an external oxidation of **A** to the cationic σ-complex **C** (Scheme 2) that rearomatizes by protonation of the Ti–O bond to liberate the desired organic product **B** as in classical Friedel–Crafts alkylations. However, in this manner, Cp_2_Ti­(IV)­Cl_2_, and not Cp_2_Ti­(III)­Cl, is generated, which needs to be reduced to form the active catalyst again.

**Scheme 2:**
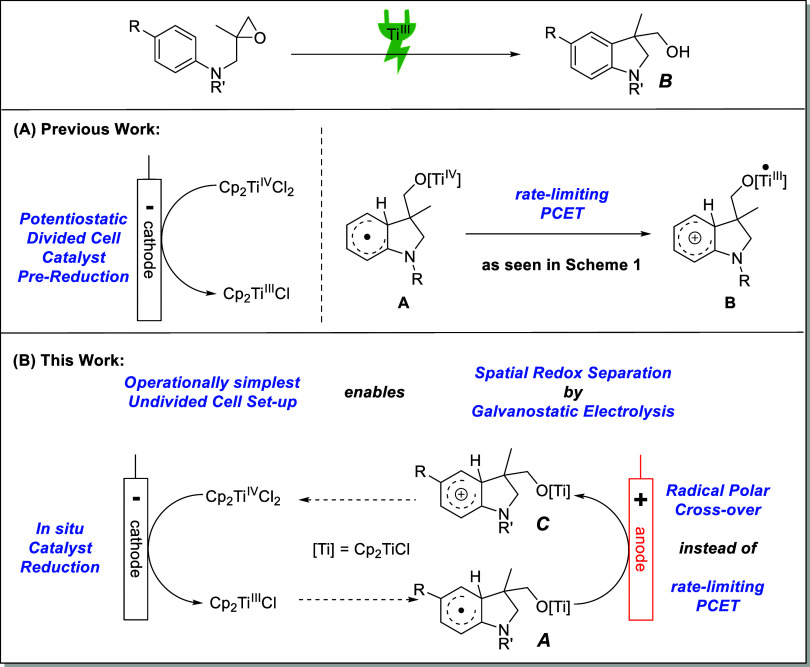
Previous Potentiostatic Prereduction of Titanium Complexes and Rate-Determining PCET (A) and Merging Titanocene Catalysis with Electro-organic Synthesis in an Undivided Cell for Resolving the Critical Issues of the Radical Arylation (B)

The resulting change in mechanism by decoupling electron from proton transfer in a radical polar crossover requires both a reduction and an oxidation step, from two different sources.^15^

An intriguing way to meet this challenge and to tap the potential of the radical polar crossover is the spatial separation of both reagents. To this end, an electrochemical reaction in an undivided cell that enforces spatially separated oxidation at the anode and reduction at the cathode is highly attractive (Scheme 2). In order to keep our setup operationally as simple as possible, we chose to perform our reaction in a galvanostatic beaker-type electrolysis cell.^11^ Noteworthy, a variety of such setups are commercially available. In contrast to this, in previous studies, Cp_2_TiX_2_ was potentiostatically prereduced. The activated solution was afterward used under reflux conditions to realize the radical arylation of epoxides. In these reactions, the rate-limiting PCET is reliant on the oxidation potential of the employed titanocene complex. For more electron-deficient substrates, Cp_2_TiCl_2_ is not a suitably oxidizing catalyst.^10^

To realize the desired radical arylation, we envisioned the electrochemical in situ reduction of Cp_2_Ti­(IV)­Cl_2_ to the active catalyst Cp_2_Ti­(III)Cl to proceed at the cathode and the oxidation of the radical σ-complex **B** to the cationic radical σ-complex **C** at the anode.

## 2 Results and Discussion

We started our investigations with the reaction of **S1** to **P1** (Table 1) that has been studied mechanistically with chemically reduced catalysts.^12e,16^ In analogy to these reactions, THF was used as solvent and Cp_2_TiCl_2_ was employed as catalyst with a loading of 10 mol %. The supporting electrolyte was Bu_4_NPF_6_ (0.2 M, 2.0 equiv with respect to the substrate), and glassy carbon electrodes were used (for details of the cell setup including a photograph, see the Supporting Information).

**Table 1:**
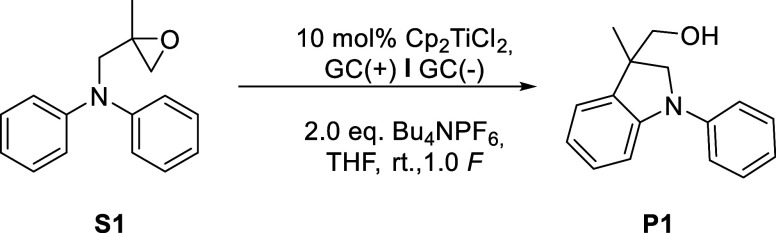
Initial Screening for the Radical Arylation of Epoxides under Galvanostatic Conditions

entry	current density, mA cm^–2^	**SuA**, mol %	consumption of **S1**	yield of **P1**
1	3.57	none	0%	0%
2	3.57	10	100%	0% (decomposition)
3^a^	0.94	10	100%	90% NMR yield 85% isolated
4	0.71	10	87%	not isolated
5	no current	10	0%	0%
6^a^ ^,^ ^b^	0.94	10	0%	0%
7^a^	0.94	none	100%	70% NMR yield

a 1.1 F.

b No Cp_2_TiCl_2_.

The critical issue of suitable reaction conditions is the interception of the radical σ-complex **A** by the anodic oxidation to the cationic σ-complex **C**, the radical polar crossover. We assumed that this oxidation would be promoted by a relatively high current density that resulted in a relatively high starting voltage (the cell potential at the onset of the electrolysis) and a small inter-electrode distance. Therefore, the reactions were run at a current density of 3.57 mA/cm^2^. For the given setup, this corresponds to a current of 10 mA, which is a typical value in organic electrosynthesis, and the overall amount of applied charge was fixed to 1.0 *F*, which corresponds to 1.0 equiv of electrons (based on organic starting material).^2^

Under the conditions investigated first [10 mol % Cp_2_TiCl_2_, 1.0 equiv of **S1** (0.1 M in THF)], only **S1** was recovered, and no formation of **P1** was observed (Table 1, entry 1). Our explanation for this finding is that no catalytically active Cp_2_TiCl was formed by cathodic reduction under these conditions. It has been demonstrated by cyclic voltammetry that for an efficient electrochemical reduction of Cp_2_TiCl_2_ to Cp_2_TiCl in THF, it is mandatory that chloride has to be abstracted from the product of the initial reduction under potentiostatic conditions. This has been achieved with the aid of reversible abstraction of chloride by supramolecular additives such as thioureas, squaramides, and sulfonamides via hydrogen bonding. Otherwise, the catalytically inactive anionic [Cp_2_TiCl_2_]^−^ is formed.^13^ In this study, we chose **SuA** as a novel additive because the analysis of the cyclic voltammograms of the electrochemical reduction of Cp_2_TiCl_2_ in THF suggests (Figure 1) that **SuA** is most efficient in abstracting chloride by reversible hydrogen bonding.^13,17^ This shifts the E_q_C_r_ equilibrium toward the formation of Cp_2_TiCl.

**Figure 1:**
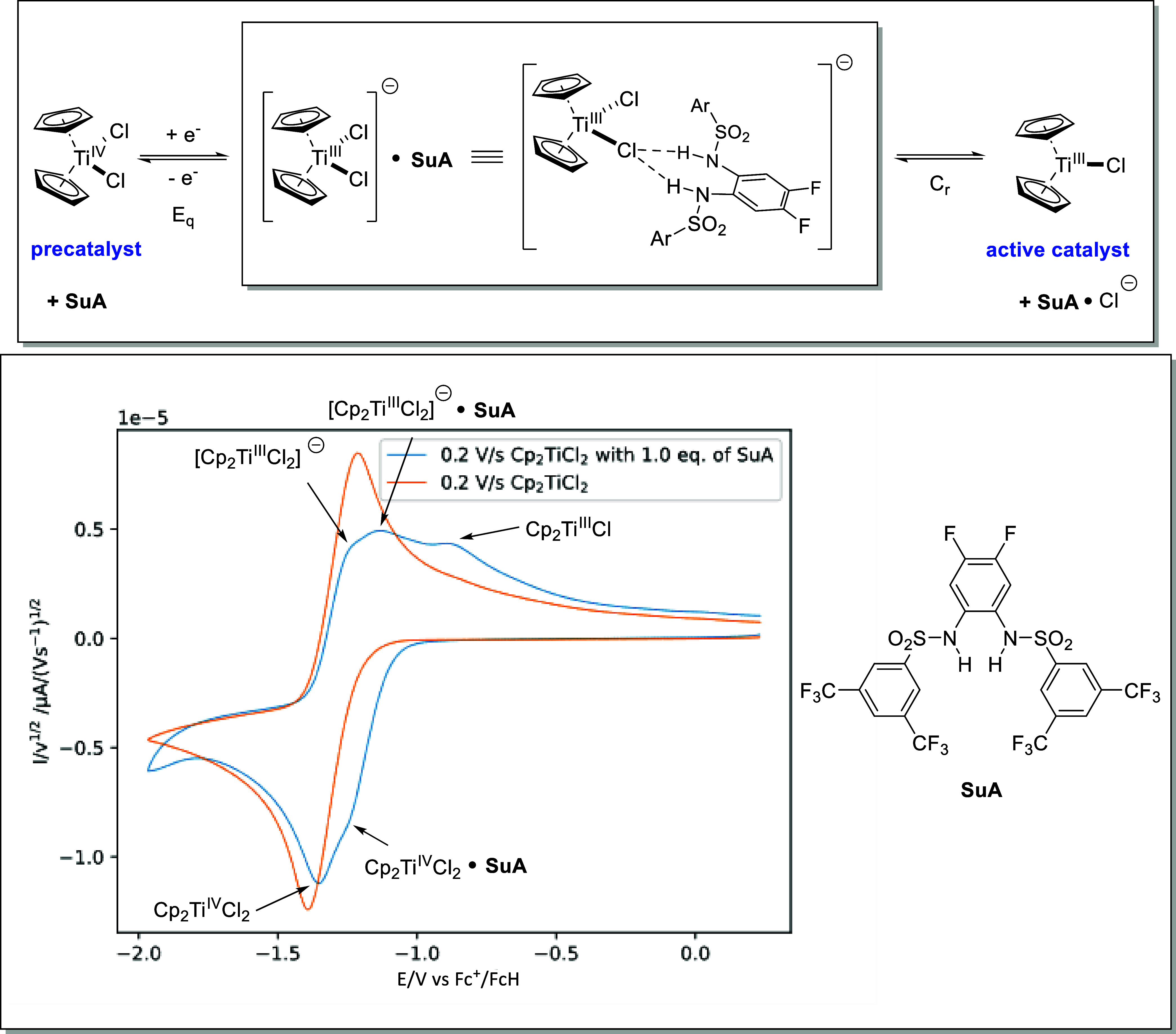
(Top) E_q_C_r_-equilibrium of the electrochemical reduction of Cp_2_TiCl_2_ in the presence of **SuA**. (Bottom) CVs of Cp_2_TiCl_2_ and Cp_2_TiCl_2_ in the presence of **SuA** (1:1) at *ν* = 0.2 V s^–1^. CVs were recorded in 0.2 M Bu_4_NPF_6_/THF. For CVs at other scan rates, see the Supporting Information.

However, while the addition of **SuA** (10 mol %) to the reaction mixture resulted in a complete consumption of **S1** as judged by ^1^H NMR analysis of the crude reaction mixture, no product formation but extensive decomposition of **S1** was observed (Table 1, entry 2). Presumably, this is caused by rapid oxidation of **S1** or, less likely, intermediates formed from **S1**.

To address this issue, we reduced the current density and thereby the cell potential to avoid extensive overoxidation. Gratifyingly, at 0.94 mA/cm^2^ that resulted in a cell potential of 2.0 V, **S1** was completely consumed and **P1** was formed in an NMR yield of 90% (measured against an internal standard, Table 1, entry 3). After workup and purification, **P1** was obtained in a very good isolated yield of 85%.

A further reduction in the current density of 0.71 mA/cm^2^ resulted in an incomplete conversion of **S1** (about 15% of the substrate remained unreacted), and no attempts to isolate the product or further optimize the current density were undertaken (Table 1, entry 4). We note, however, that without current (Table 1, entry 5), or without Cp_2_TiCl_2_ (Table 1, entry 6), no **P1** was formed. Finally, we investigated the paired electrolysis at a current density of 0.94 mA/cm^2^ in the absence of **SuA**. In this case, we observed a 70% NMR yield of **P1** together with some decomposition product deposited at the anode (Table 1, entry 7). Compared to the electrolysis in the presence of **SuA**, the cell potential of 2.4 V was higher by 0.4 V. We attribute this significant increase to an increased formation of the catalytically inactive [Cp_2_TiCl_2_]^−^. This results in a lower catalyst concentration and a slower reaction with lower mass transport. Because of the higher cell potential, larger amounts of decomposition products are formed.

With these results in hand, we shifted our focus toward substrate **S2**. With chemically or electrochemically reduced Cp_2_Ti­(IV)­Cl_2_, **P2** is typically formed in low amounts (<15%) or not at all, because the titanocene is not oxidizing enough to enable the PCET for rearomatization of the less electron-rich σ-complex **A** (Scheme 1).^10,12,13^ Therefore, **S2** is ideally suited to demonstrate the relevance of the oxidative radical polar crossover at the anode in our undivided cell setup (Table 2).

**Table 2:**
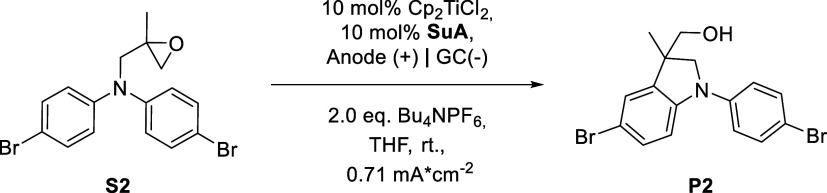
Identification of Optimal Reaction Conditions for the Synthesis of P2

entry	amount of applied charge	anode material	consumption of **S2**	result
1	1.0 *F*	GC	25%	not isolated
2	1.0 *F*	RVC^a^ 100 ppi	100%	decomposition
3	1.0 *F*	Pt	100%	decomposition
4^b^	2.5 *F*	GC	100%	81% yield

a RVC = reticulated vitreous carbon (ppi = pores per inch).

b Current density: 0.94 mA cm^–2^.

Under the optimized conditions for **S1**, **S2** shows a conversion of only 25% (Table 2, entry 1). Since the reduction of Cp_2_TiCl_2_ in combination with **SuA** on GC under galvanostatic conditions appears to be unproblematic, we decided to change the anode material to study the influence of the postulated oxidative radical polar crossover. An increase in the surface area using reticulated vitreous carbon (RVC) (Table 2, entry 2) or a material change to Pt (Table 2, entry 3) did not result in product formation, and complete decomposition of the substrate was observed. However, a significant increase in the amount of applied charge (2.5 F) gave full conversion to **P2**, which could be isolated in 81% yield. The required increase in the amount of applied charge can be readily explained by the anodic oxidation of Cp_2_Ti­(III)Cl that outruns the desired radical polar crossover. In this manner, our reaction consumes additional charge by shuttling electrons between the electrodes through the Cp_2_Ti(III)Cl/Cp_2_Ti(IV)Cl_2_ redox pair.

To check the generality of our reaction, we investigated its substrate scope, as summarized in Scheme 3. In addition to **P1** and **P2**, the methyl-substituted **P3** and fluorinated **P4** were obtained in good yields. **P4** could not previously be obtained using titanocene catalysts.^10,12^ Most notably, the −CF_3_-substituted **P5** can only be synthesized under our galvanostatic conditions. This finding highlights the power of the electrochemical approach. The *N*-methylated products **P6** and **P7**, which are prone to decomposition by amine oxidation, can nevertheless be obtained in satisfying yields.^18^ This highlights the chemoselectivity of the electrochemical reaction conditions.

**Scheme 3:**
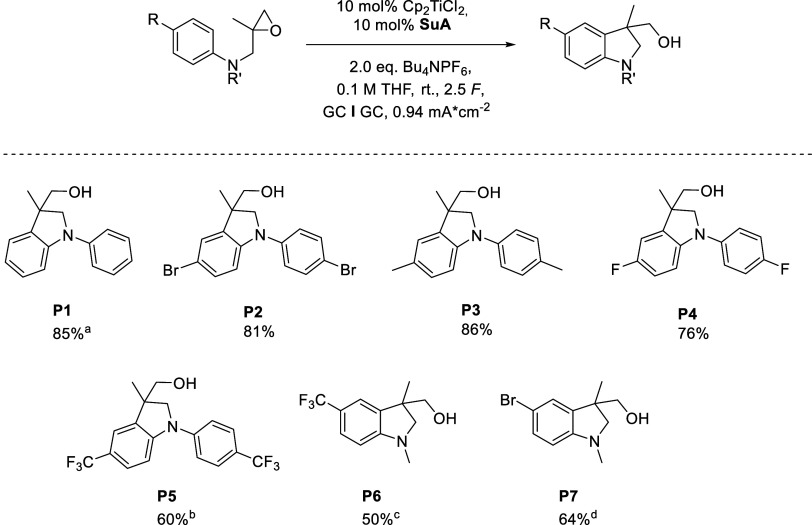
Substrate Scope of the Radical Arylation of Epoxides under Galvanostatic Conditions

a 1.1 *F*.
b 1.0 *F*.
c 1.5 *F*.
d 3.5 *F*.

### 2.1 Substituent Effects as Evidence of the Radical Polar Crossover

The mechanistic key aspect of our reaction is the oxidation of the radical σ-complex **A** to its cationic equivalent **B**. The oxidation of **A** is more difficult with electron-withdrawing substituents and, hence, under galvanostatic conditions, a distinct effect of the substrate on the cell potential must be expected that can be used as a mechanistic probe for the radical polar crossover. The effect of the anodic oxidation on the cell potential is the determining factor and indicative of substituent effects. A decreased electron-donating character of the arenes’ substituents results in an increased oxidation potential of **A**. The cathodic potential for the reduction of Cp_2_TiCl_2_, on the other hand, remains unchanged. Therefore, the overall observed cell potential in the galvanostatic setup should critically depend on the substrate. To verify if this is indeed the case and to quantify the correlation between the substituents and the cell potential, we examined the starting voltage (cell potential directly at the onset of the electrolysis) against the Hammett constants of the p-substituents.

As shown in Figure 2, a linear correlation between log­(*V*_R_/*V*_H_) and the substituents’ Hammett constants σ_p_ is observed. The relatively small slope observed should be indicative of little charge built up in the rate-determining step, which is the case for the proposed mechanism. Moreover, the starting voltage correlates well with the Hammett parameter σ_p_ of the respective substituents (Figure 2).^19^ The slope indicates that the inductive effect of the substituents used does have the expected influence on the mesomeric stabilization of the cationic σ-complex. However, stabilization by the *N*-alkyl group remains relevant.

**Figure 2:**
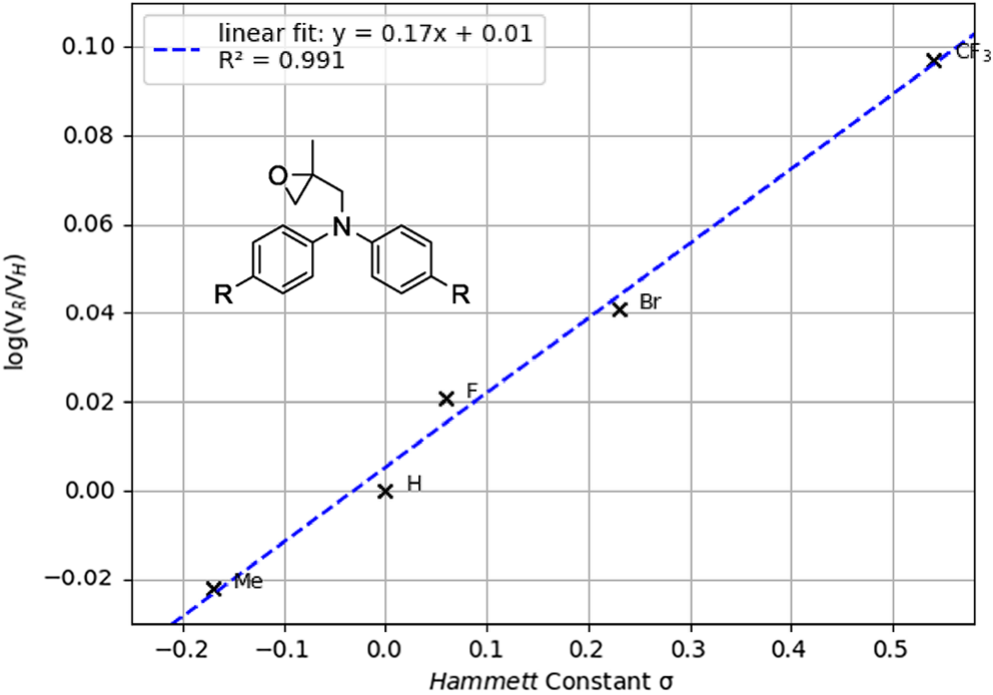
log­(*V*_R_/*V*_H_) versus Hammett constants of the p-substituents.

This suggests that the expected radical polar crossover is indeed possible and that the ensuing rearomatization of the cationic σ-complex formed by protonation of the Ti–O bond is fast.^12e,16^ A reaction proceeding via PCET as in the classical titanocene-catalyzed radical arylation can be ruled out.

However, a hydrogen atom transfer (HAT) from the radical σ-complex **A** to other radical species remains a mechanistic alternative for the rearomatization of **A**. To assess this possibility, we investigated the potential oxidation of chloride released in the reduction of Cp_2_TiCl_2_ and that of THF, our solvent. CV experiments of the reaction mixture of **S2** and **S5** did not show any wave pertaining to uncomplexed (‘free’) chloride (for CV measurements, see the Supporting Information). This can be attributed to the halide-binding ability of **SuA**, which is essential (in stoichiometric amounts relative to Cp_2_TiCl_2_) for Cl^–^ abstraction from the electrochemically generated [Cp_2_TiCl_2_]^−^ under our conditions. Sulfonamides have previously been shown to be potent halide binders in titanocene catalysis.^10b^ In this study, it was shown that even with a weaker sulfonamide binder^17^ (analogous to **SuA**, but without the −F substituents on the central arene), no free chloride is present in solution. Since **SuA** is an even stronger Cl^–^ binder, this will also be the case with **SuA**. Therefore, a contribution of a chloride radical in a HAT reaction with **A** is unlikely.

Oxidation of THF only occurs at a higher potential than the oxidation of the most deficient **S5**. Thus, a role of oxidation products of THF, such as THF^•+^, in the rearomatization can be safely ruled out (for CV measurements, see the Supporting Information). As a consequence, we deem a HAT as a mechanism for the rearomatization of the by anodically generated radical species as rather improbable.

Based on this analysis and the (over) stoichiometric amount of applied charge *F* required for all substrates, we propose a mechanism of the titanocene-catalyzed radical arylation (Scheme 4) featuring both cathodic electrochemical catalyst reduction and anodic electrochemical oxidation of the radical σ-complex **A** that results in a radical polar crossover. In this manner, the limitation of the classical arylation, the rate-determining PCET step (Scheme 1), can be circumvented by decoupling electron transfer and proton transfer to increase the substrate scope of the reaction.

**Scheme 4:**
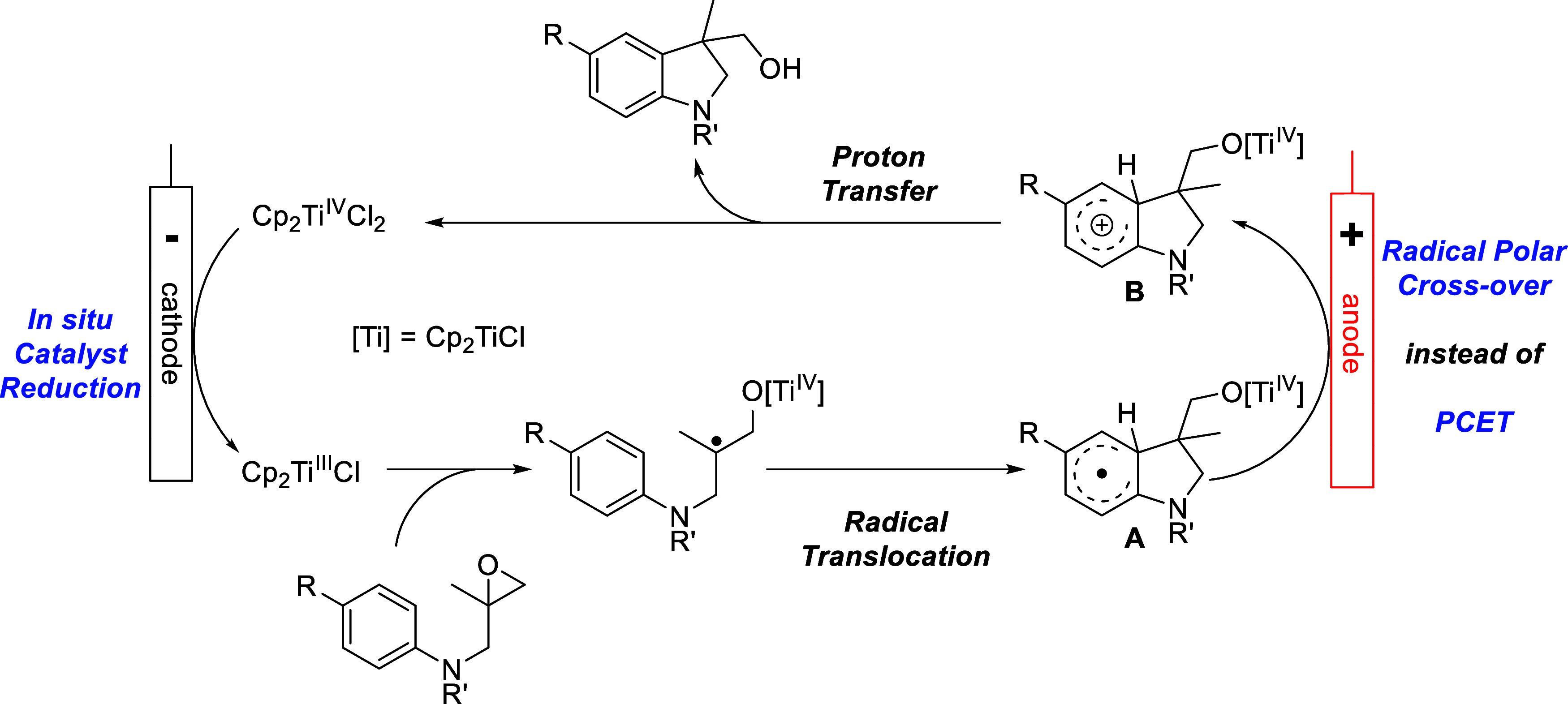
Postulated Mechanism for the Radical Arylation of Epoxides Assisted by a Consecutive Paired Electrolysis

Counter reactions in electrosynthesis often represent a critical point for realization, as they need to run in tandem with the desired reaction without interfering with product generation.^20^ In our system, however, it seems that both electrode reactions serve in a productive cooperative manner. This may be considered an efficient consecutive paired electrolysis.

To provide further evidence in support of this notion, we investigated the substrates **S1**, **S2**, and **S5** in a divided cell setup (Table 3). Those substrates were chosen based on their varying electronic demand, allowing for a comparison between the classical PCET, only feasible for **S1** with Cp_2_TiCl_2_, and the radical polar crossover postulated here. To this end, the substrate and catalyst were placed in the cathodic half-cell. In this manner, no interaction of these species with the anode is possible and, as a consequence, neither the critical anodic electrochemical oxidation of the radical σ-complex nor the anodic oxidation of the active Ti­(III) catalyst is possible.

**Table 3:**
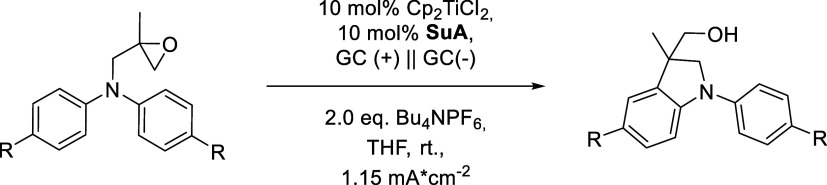
Control Experiments in a Divided Cell Setup

entry	substrate	amount of applied charge	consumption of substrate	yield of product^a^
1	**S2**	0.1 F	4%	0%
2	**S2**	2.5 F	100%	decomposition
3	**S5**	0.1 F	0%	0%
4	**S5**	1.0 F	40%	decomposition
5	**S1**	0.1 F	78%	54%
6	**S1**	1.1 F	87%	decomposition

a NMR yield.

In the presence of chemically or electrochemically generated Cp_2_TiCl, **S2** and **S5** cannot be transformed to **P2** and **P5** because the PCET (Scheme 1), leading to the rearomatization of the radical σ-complex is not possible. This is also reflected by the experiments in the divided cell setup, where the amount of applied charge (0.1 F) is only sufficient for catalyst reduction. No product formation was observed (Table 3, entries 1 and 3). For a purely catalyst-controlled reaction, an additional amount of applied charge (above 0.1 F) should not benefit the productive conversion of the substrates in a divided cell. Indeed, increasing the amount of applied charge leads to extensive decomposition of the substrates (Table 3, entries 2 and 4). In the undivided cell setup, both **P2** and **P5** could be obtained in satisfactory yields.

The situation with **S1** is different, since **P1** can be obtained with chemically or electrochemically reduced Cp_2_TiCl because the radical σ-complex can be oxidized by PCET.^12d^ This is also the case in the divided cell setup when Cp_2_TiCl_2_ is reduced by applying catalytic amounts of applied charge. However, **P2** is formed in a rather low NMR yield of only 54% (Table 3, entry 5). Once again, with 1.1 *F* of applied charge, extensive decomposition of the substrate was observed (Table 3, entry 6). In the undivided cell, an isolated yield of 85% of **P1** was obtained.

These control experiments clearly demonstrate that in the undivided single-cell setup, the oxidation of the radical σ-complex at the anode is essential for an efficient radical arylation. Therefore, all evidence suggests that our process should indeed be considered as an efficient consecutive paired electrolysis.

Examples of this type of reactivity are reported quite rarely.^21^ Controlling and driving the same transition-metal catalyst by both electrodes is the first of its kind to the best of our knowledge.

## 3 Conclusion

In conclusion, we have combined the mildness of transition-metal-catalyzed radical chemistry with the sustainability of electro-organic synthesis. By utilizing an undivided cell under a galvanostatic setup, the rate-determining oxidation of the radical σ-complex by a PCET is circumvented by anodic oxidation of the radical σ-complex to its cationic analogue, while catalyst activation occurs by sulfonamide-assisted cathodic reduction of Cp_2_TiCl_2_. This successful radical polar crossover results in a highly unusual consecutive paired electrolysis. Moreover, our approach allows an increase in the substrate scope of the titanocene-catalyzed radical arylation and circumvents the elaborate catalyst screening with more electron-deficient titanocenes. All electrosynthetic prerequisites are met for scale-up in batch-type or flow electrolysis. Furthermore, our work underscores that combinations of transition-metal catalysts and electrosynthesis can provide advantages beyond facilitated preparation of active species by enabling alternative reaction mechanisms.

## Supplementary Material



## Data Availability

The data underlying this study are available in the published article and its Supporting Information.

## Notes

Further experimental details and full characterization of the generated compounds (PDF)

## Notes

In the memory of Prof. Dr. Manfred T. Reetz.
