# Supplementary material for: Generalization of Titanocene-Catalyzed Arylation of Epoxides through Radical Polar Crossover by Consecutive Paired Electrolysis
Source: JACS Au. 2026 Jun 11;6(7):3694–702. doi: 10.1021/jacsau.6c00215 (PMC13417293; doi:10.1021/jacsau.6c00215)
Supplement: Supplementary file 1 [file au6c00215_si_001.pdf]

# Generalization of Titanocene Catalyzed Arylation of Epoxides through Radical Polar Crossover by Consecutive Paired Electrolysis

Supporting Information

Niklas Schmickler,[a] Darryl F. Nater,[b] Siegfried R. Waldvogel*,[b,c] and Andreas Gansäuer*[a]

[a] N. Schmickler, Prof. Dr. A. Gansäuer, Kekulé-Institut für Organische Chemie und Biochemie Universität Bonn Gerhard-Domagk-Straße 1, 53121 Bonn, Germany.

[b] Dr. D. F. Nater, Prof. Dr. S. R. Waldvogel Max-Planck-Institute for Chemical Energy Conversion, Department of Electrosynthesis Stiftstraße 34–36, 45470 Mülheim an der Ruhr, Germany.

[c] Prof. Dr. S. R. Waldvogel, Karlsruhe Institute of Technology, Institute of Biological and Chemical Systems – Functional Molecular Systems (IBCS-FMS), Kaiserstraße 12, 76131 Karlsruhe, Germany.

Correspondence to: Prof. Dr. A. Gansäuer, andreas.gansaeuer@uni-bonn.de or Prof. Dr. S. R. Waldvogel, siegfried.waldvogel@cec.mpg.de

1. General information ........................................................ S1

2. Reagents ........................................................ S2

3. General procedures ........................................................ S3

3.1. Electrochemical setup ........................................................ S3

3.2. General protocol for the radical arylation of epoxides under galvanostatic conditions (GP1) 4

4. CV experiments ........................................................ S4

5. Synthesis of the compounds ........................................................ S12

5.1. Substrate synthesis ........................................................ S12

5.2. Titanocene catalyzed radical arylation under electrochemical conditions ........................ S14

6. Spectra ........................................................ S21

7. References ........................................................ S30

## 1. General information

All moisture- or oxygen-sensitive reactions were carried out under inert atmosphere (Ar) using a Glovebox (MBraun Lab Master 130). The solvents used were purified M Braun MB-SPS-800 system (THF). Other solvents were used as p.a. grade. All reactions were monitored by thin-layer chromatography (TLC) on Merck silica gel 60 $F_{254}$ plates using UV light as visualizing agent. Preparative column chromatography was either performed manually by flash column chromatography on Merck silica gel 60 (0.035–0.070 mm) or using prepacked puriFlash™ silica columns (PF-15SIHP-F0040 Interchim, Montlucon Cedex, France) using a puriFlash™-system (puriFlash™ XS520 Plus, Interchim Montlucon Cedex, France) with an integrated UV detector. $^{1}H$, $^{13}C$ NMR spectra were recorded on Bruker Avance I 400 MHz ($^{1}H$-base frequency: 400.13 MHz), Bruker Avance I 500 MHz ($^{1}H$ base frequency: 499.13 MHz), or Bruker Avance III HD Ascend 500 MHz ($^{1}H$-base frequency: 500.13 MHz) at 298 K. Chemical shifts are denoted in ppm (δ), and calibrated by using residual nondeuterated solvent $C_6H_6$ (7.16 ppm) as internal reference for $^{1}H$ NMR and $C_6D_6$ (128.1 ppm) as internal reference for $^{13}C$ NMR. NMR yields were determined by integration against $CH_2Br_2$. High resolution mass spectra analysis was performed on a Thermoquest MAT 95 XL instrument (Thermo Finnigan, EI). IR spectra were recorded on the 380 or Shimadzu IRSpirit.

## 2. Reagents

4-Fluoro-*N*-methyl-*N*-((2-methyloxiran-2-yl)methyl)aniline (**S6**) was obtained from *Zhang* according to the reported procedure.[1]

(**E1**), (**S1**), (**S2**), (**S3**), (**S4**) and (**S7**) were synthesized according to the reported protocols.[2]

**SuA** was synthesized according to the reported procedure.[3]

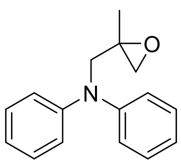

**S1**

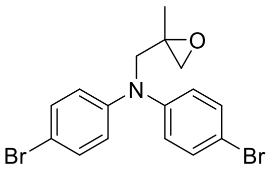

**S2**

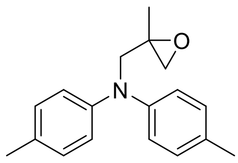

**S3**

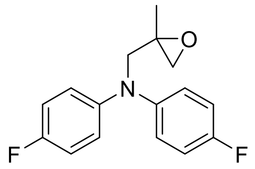

**S4**

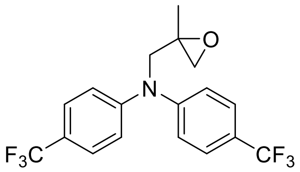

**S5**

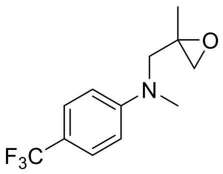

**S6**

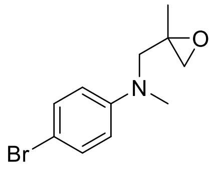

**S7**

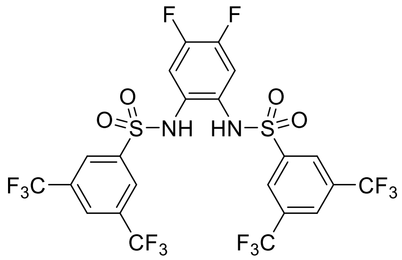

**SuA**

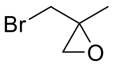

**E1**

# 3. General procedures

## 3.1. Electrochemical setup

**Undivided Cell**

Electrochemical reactions were carried out using a multichannel galvanostat HMP4040 (Rohde & Schwarz, München, Germany) in an undivided glass cell with a volume of 5 mL equipped with two electrodes (interelectrode gap: 8 mm) and a cross-shaped stirring bar. The electrode materials (GC and RVC: 8 mm x 55 mm x 2 mm) and (Pt: 10 mm x 70 mm x 0.1 mm) were obtained and used as stated in the table below.

| Entry | Electrode Material | Specification | Supplier |
|---|---|---|---|
| 1 | Glassy Carbon (GC) | Sigradur® | HTW Hochtemperatur Werkstoffe GmbH |
| 2 | Reticulated vitreous carbon (RVC) | 100 ppi, Duocel® | ERG Aerospace Corporation |
| 3 | Platinum | >99% | ÖGUSSA |

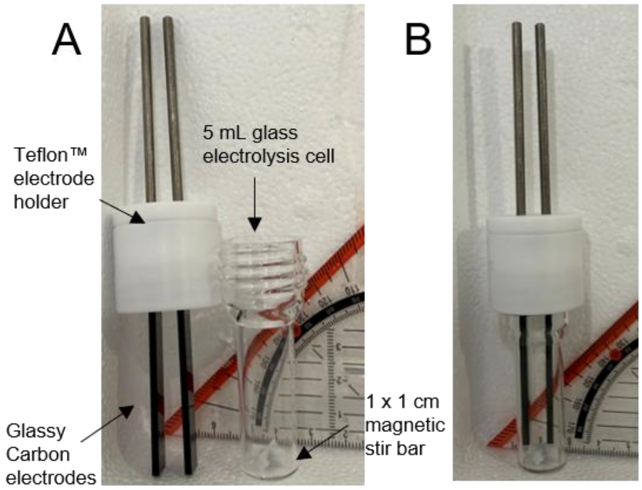


Figure S1: Beaker-type undivided glass cell used.

**Divided Cell**

Electrochemical reactions were carried out using a multichannel galvanostat HMP4040 (Rohde & Schwarz, München, Germany) in a divided Teflon™ cell with a volume of 5 mL each and equipped with a round stirring bar. A porous glass frit (P4) was used as a separator, sealed by an EPDM ring. Glassy carbon electrodes (GC: 10 mm x 70 mm x 3 mm) were used as cathode and anode material, which was obtained as stated below.

| Entry | Electrode Material | Specification | Supplier |
|---|---|---|---|
| **1** | Glassy Carbon (GC) | Sigradur® | HTW Hochtemperatur Werkstoffe GmbH |

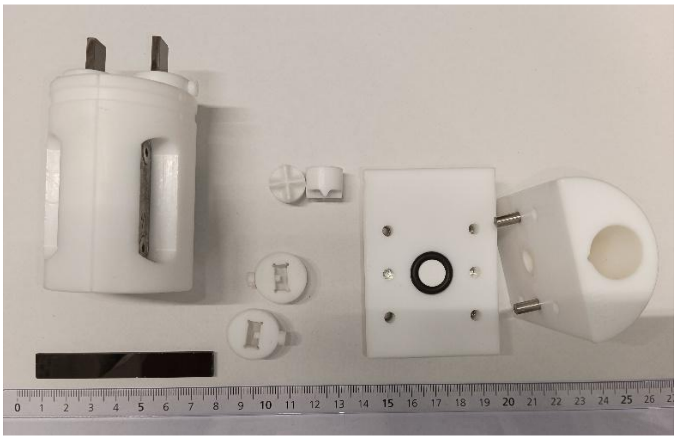

Figure S2: Divided Teflon™ cell used.

### 3.2. General protocol for the radical arylation of epoxides under galvanostatic conditions (GP1)

$Cp_2TiCl_2$ (0.10 eq., 12.5 mg, 0.05 mmol), **SuA** (0.10 eq., 35 mg, 0.05 mmol), $Bu_4NPF_6$ (2.00 eq., 387 mg, 1.00 mmol) and the corresponding substrate (1.00 eq., 0.50 mmol) were placed in an undivided beaker-type glass cell and put inside of a *Glovebox*. THF (5 mL) was added and the mixture was stirred for three minutes at setting 7 on an IKA RCT basic magnetic stirrer. The lid including the corresponding electrodes was attached to the cell, so that the electrodes were immersed 4 cm deep into the solution. The amperage was set so that a current density of 0.91 mA*$cm^{-2}$ (3 mA for this set-up) resulted and the amount of applied charge was set to 2.5 *F* (120 C using 0.5 mmol of substrate). The electrolysis was conducted at room temperature under constant stirring. After completion the cell was removed from the *Glovebox* and the electrodes were rinsed with MTBE. The conducting salt was filtered off, the crude product was loaded onto Celite™ and purified by automated flash column chromatography (CH/EA) with an appropriate gradient.

## 4. CV experiments

**CV of the catalyst**

An oven dried cyclic voltammetry cell was equipped with magnetic stir bar and filled with 10 mL freshly distilled THF. After dissolving the conducting salt $NBu_4PF_6$ (0.775 g, 0.200 mmol) background measurements were conducted to subtract the coulomb current in the analysis from the CVs recorded with analyte. $Cp_2TiCl_2$ (0.02 mmol, 5 mg) was added to the cell and CVs were recorded. The CV experiment was performed at different sweep rates (0.05 $Vs^{-1}$, 0.1 $Vs^{-1}$, 0.2 $Vs^{-1}$, 0.5 $Vs^{-1}$, 1 $Vs^{-1}$, 2 $Vs^{-1}$, 5 $Vs^{-1}$ and 10 $Vs^{-1}$) and the solution was stirred after the respective sweep rate. **SuA** (0.02 mmol, 14

mg) was added to the cell and CVs were recorded. The CV experiment was performed at different sweep rates (0.05 $Vs^{-1}$, 0.1 $Vs^{-1}$, 0.2 $Vs^{-1}$, 0.5 $Vs^{-1}$, 1 $Vs^{-1}$, 2 $Vs^{-1}$, 5 $Vs^{-1}$ and 10 $Vs^{-1}$) and the solution was stirred after the respective sweep rate. For recording the potential of FcH/$FcH^+$ redox couple a small amount of ferrocene (0.02 mmol) was added as an internal reference at the end of the experiment.

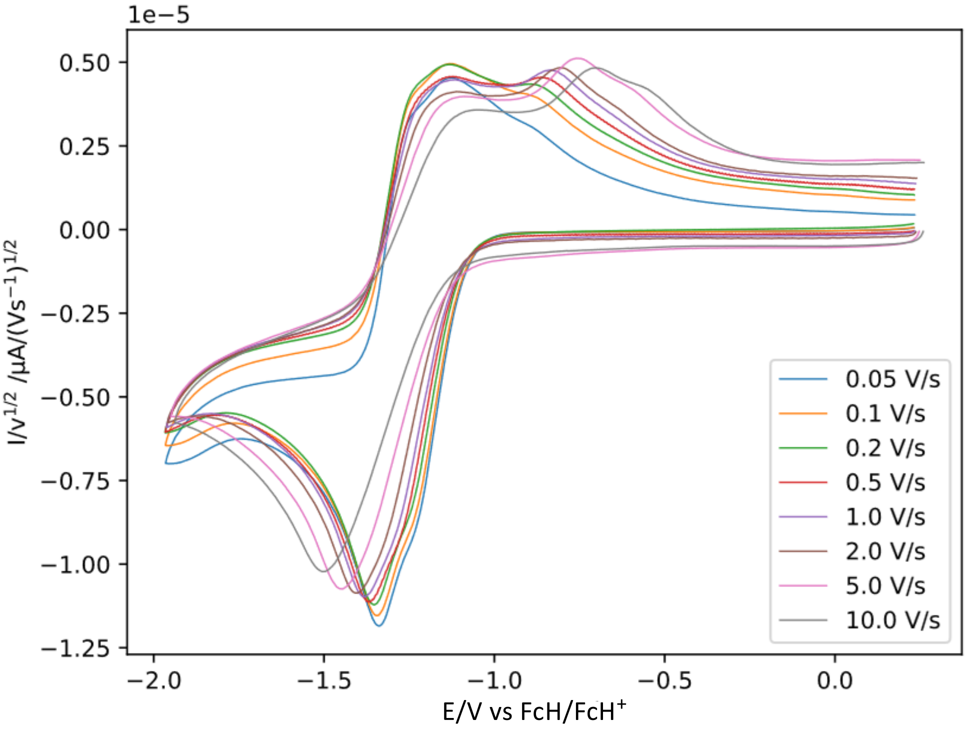


Figure S3: CV of $Cp_2TiCl_2$ with 1.0 eq. **SuA** at different scan rates.

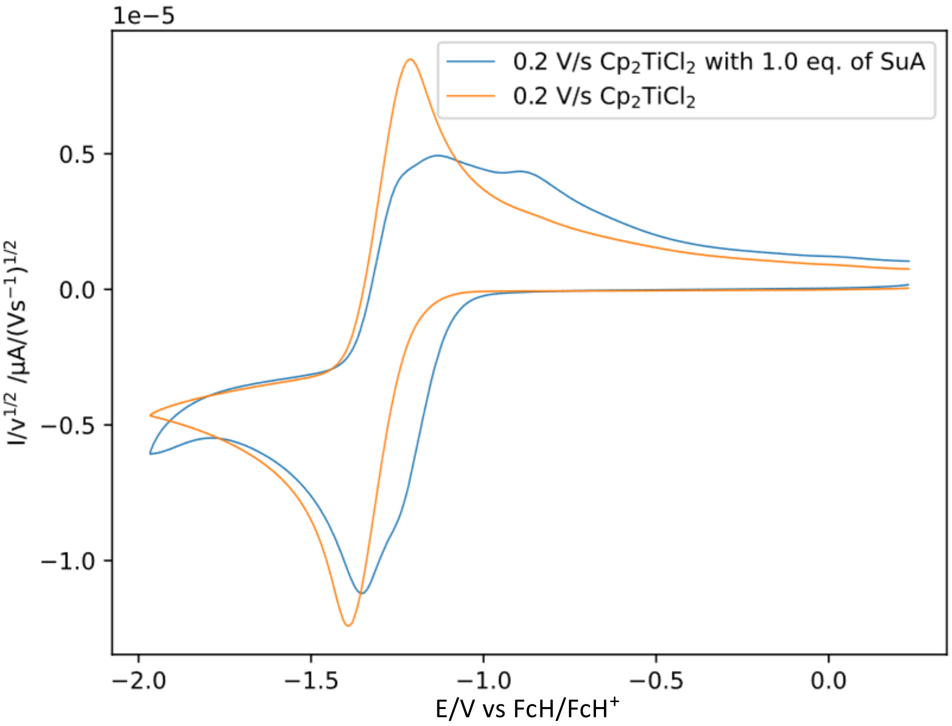


Figure S4: Comparison between $Cp_2TiCl_2$ with and without 1.0 eq. of **SuA** at a sweep rate of 0.2 V/s.

**CV control experiments**

**CV of $NBu_4Cl$**

An oven dried cyclic voltammetry cell was equipped with a magnetic stir bar and filled with 10 mL freshly distilled THF. After dissolving the conducting salt $NBu_4PF_6$ (0.775 g, 0.200 mmol) background measurements were conducted to subtract the coulomb current in the analysis from the CVs recorded with analyte. $Bu_4NCl$ (0.02 mmol, 6 mg) was added to the cell and CVs were recorded. The CV experiment was performed at different sweep rates (0.05 $Vs^{-1}$, 0.1 $Vs^{-1}$, 0.2 $Vs^{-1}$, 0.5 $Vs^{-1}$, 1 $Vs^{-1}$, 2 $Vs^{-1}$, 5 $Vs^{-1}$ and 10 $Vs^{-1}$) and the solution was stirred after the respective sweep rate. For recording the potential of $FcH/FcH^+$ redox couple a small amount of ferrocene (0.02 mmol) was added as an internal reference at the end of the experiment.

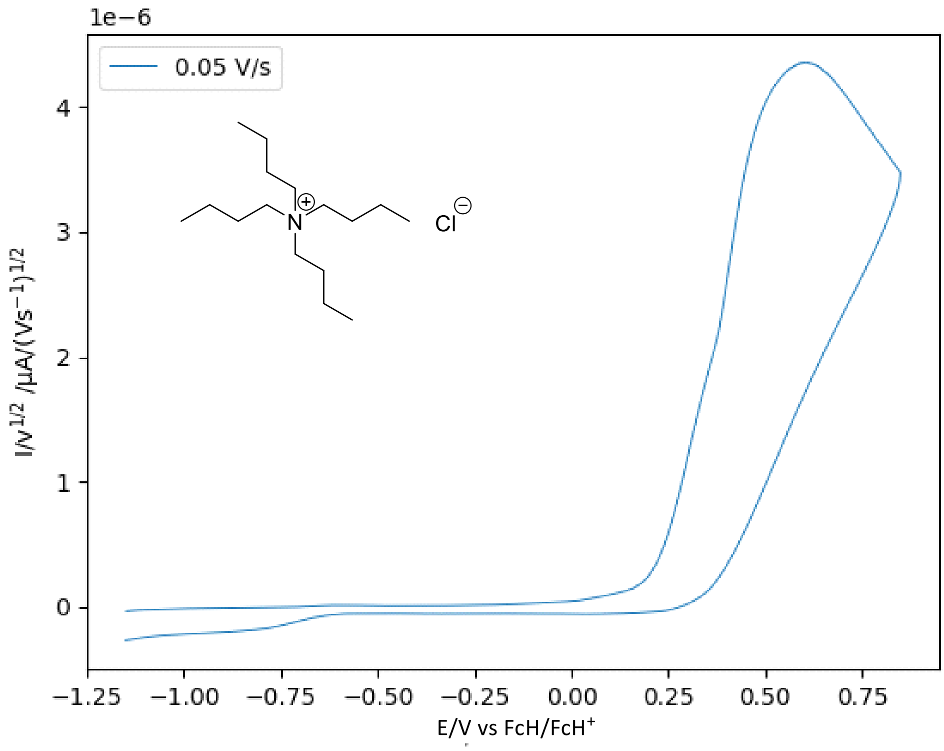


Figure S5: CV of $NBu_4Cl$ at a sweep rate of 0.05 V/s.

**CVs of S2 with $Cp_2TiCl_2$ and SuA**

An oven dried cyclic voltammetry cell was equipped with a magnetic stir bar and filled with 10 mL freshly distilled THF. After dissolving the conducting salt $NBu_4PF_6$ (0.775 g, 0.200 mmol) background measurements were conducted to subtract the coulomb current in the analysis from the CVs recorded with analyte. **S2** (0.02 mmol, 8 mg) was added to the cell and CVs were recorded. The CV experiment was performed at a sweep rate of 0.05 V/s. **SuA** (0.02 mmol, 14 mg) and $Cp_2TiCl_2$ (0.02 mmol, 6 mg) were added and the mixture was stirred until full dissolution of the catalyst. Another CV was then recorded at a sweep rate of 0.05 V/s. For recording the potential of FcH/FcH$^+$ redox couple a small amount of ferrocene (0.02 mmol) was added as an internal reference at the end of the experiment.

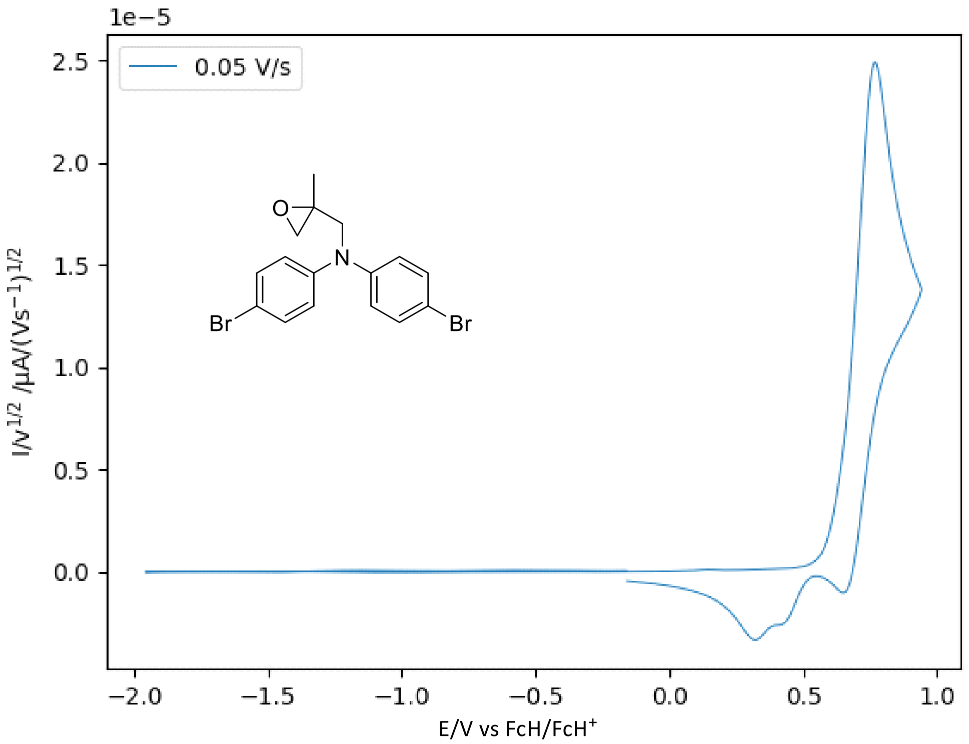


Figure S6: CV of **S2** at a sweep rate of 0.05 V/s.

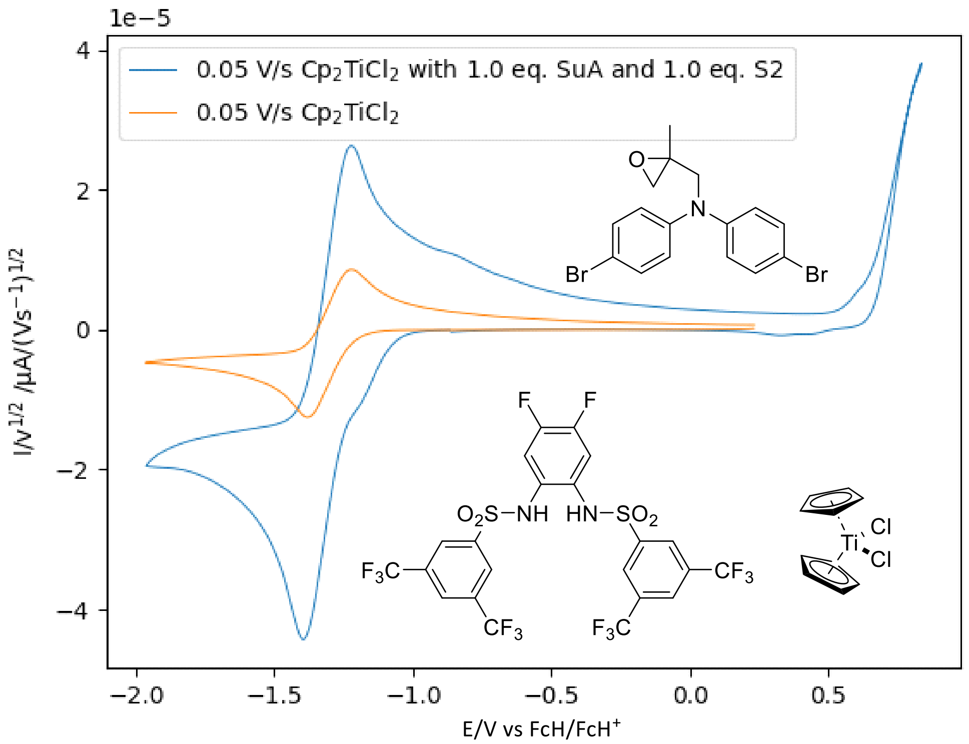


Figure S7: CV of **S2** with $Cp_2TiCl_2$ and **SuA** at a sweep rate of 0.05 V/s.

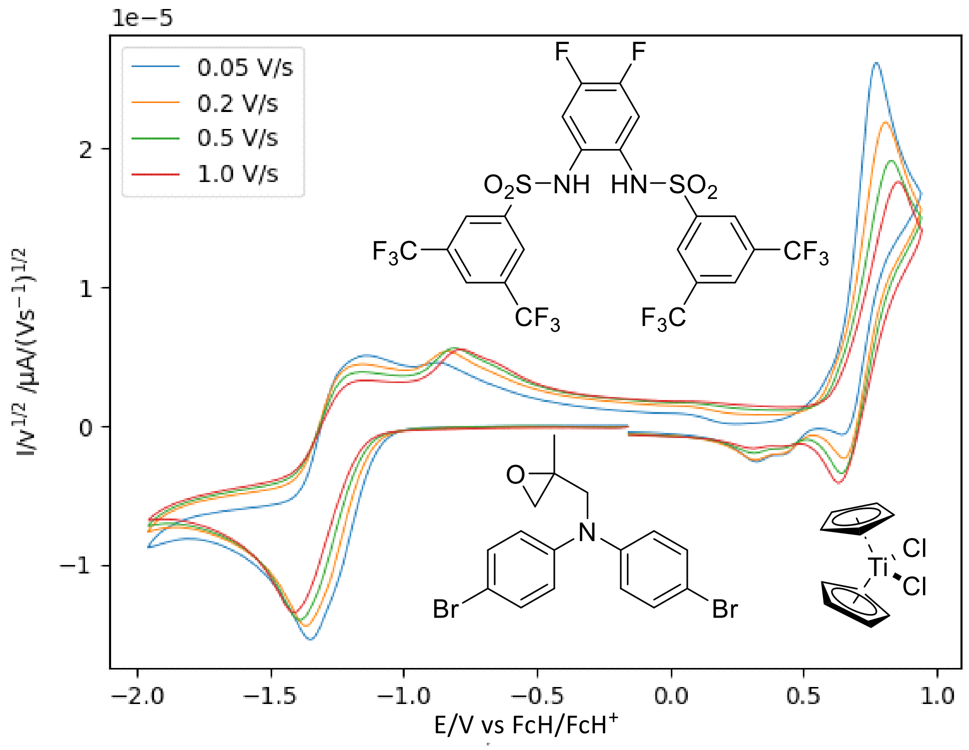


Figure S8: CV of **S2** with $Cp_2TiCl_2$ and **SuA** at a different sweep rates.

**CV of the most electron demanding substrate S5**

An oven dried cyclic voltammetry cell was equipped with a magnetic stir bar and filled with 10 mL freshly distilled THF. After dissolving the conducting salt $NBu_4PF_6$ (0.775 g, 0.200 mmol) background measurements were conducted to subtract the coulomb current in the analysis from the CVs recorded with analyte. **S5** (0.02 mmol, 8 mg) was added to the cell and CVs were recorded. The CV experiment was performed at a sweep rate of 0.05 V/s. **SuA** (0.02 mmol, 14 mg) and $Cp_2TiCl_2$ (0.02 mmol, 6 mg) were added and the mixture was stirred until full dissolution of the catalyst. Another CV was then recorded at a sweep rate of 0.05 V/s. For recording the potential of $FcH/FcH^+$ redox couple a small amount of ferrocene (0.02 mmol) was added as an internal reference at the end of the experiment.

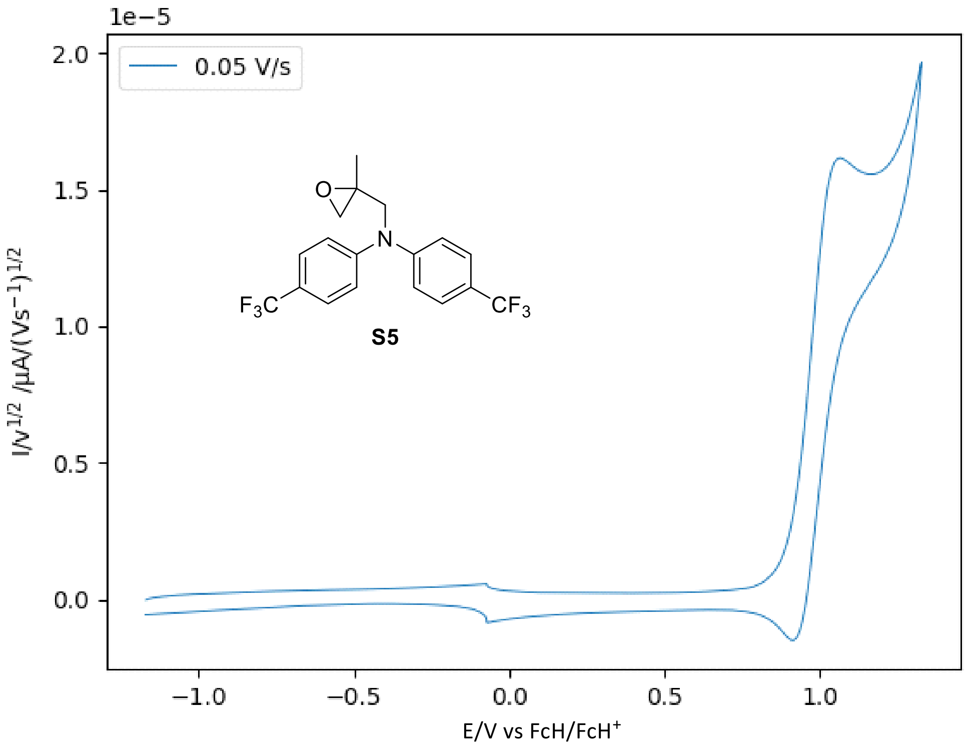


Figure S9: CV of **S5** at a sweep rate of 0.05 V/s (the increase at the upper limit of the CV can be attributed to the oxidation of THF).

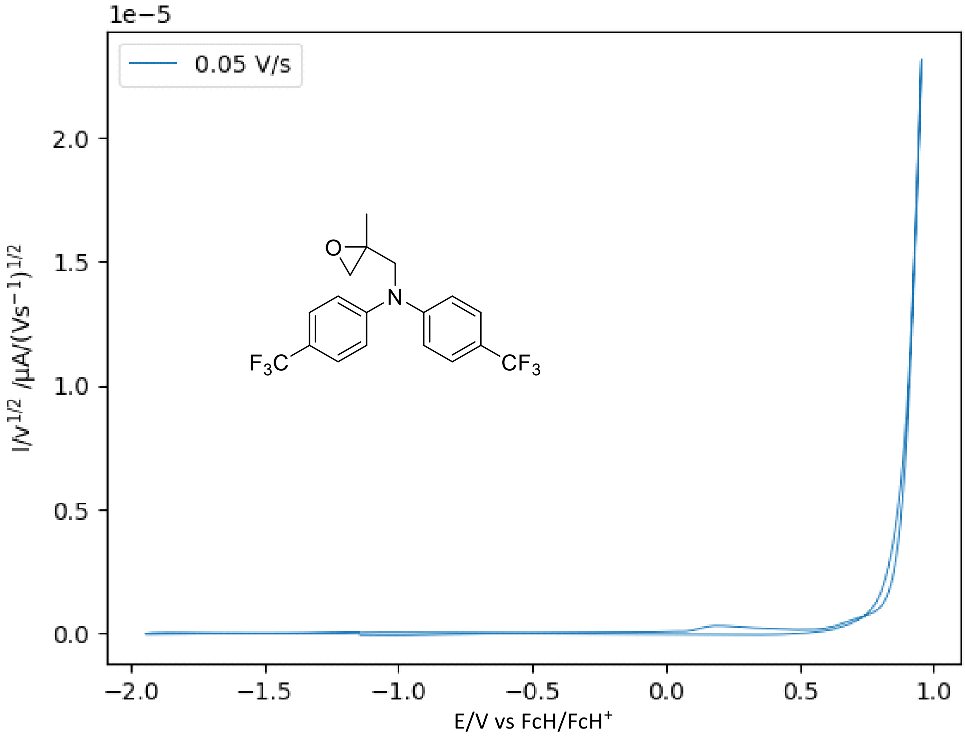


Figure S10: CV of **S5** at a sweep rate of 0.05 V/s.

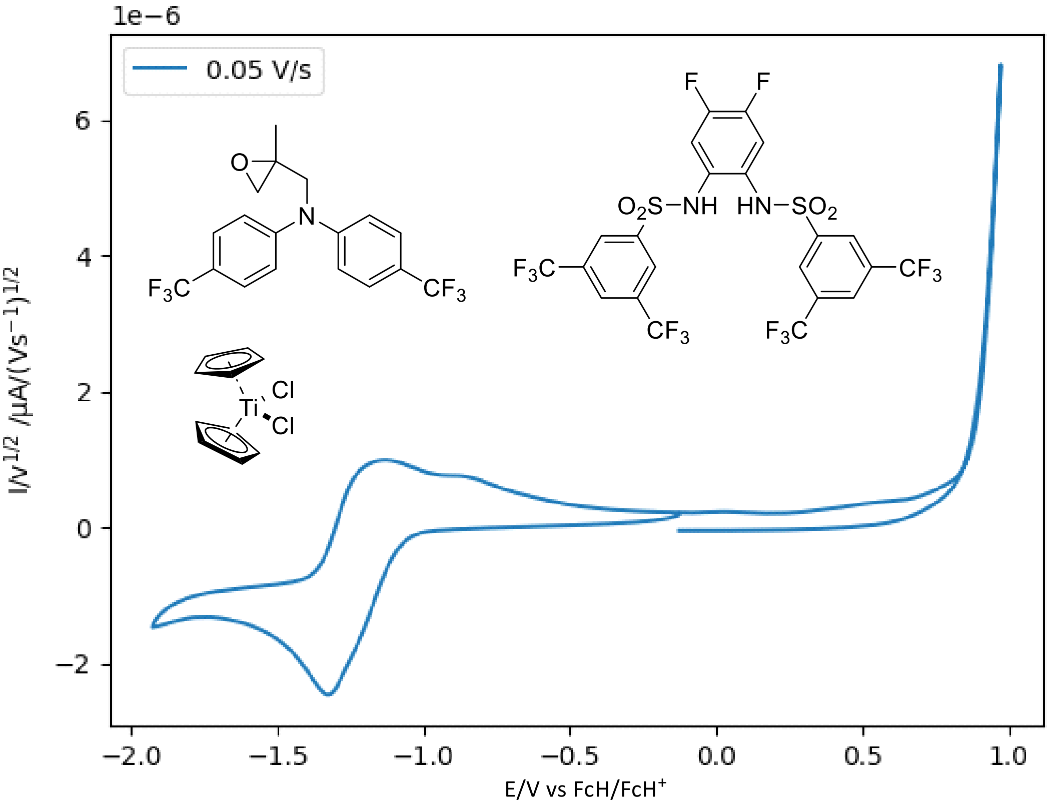


Figure S11: CV of **S5** with $Cp_2TiCl_2$ and **SuA** at a sweep rate of 0.05 V/s.

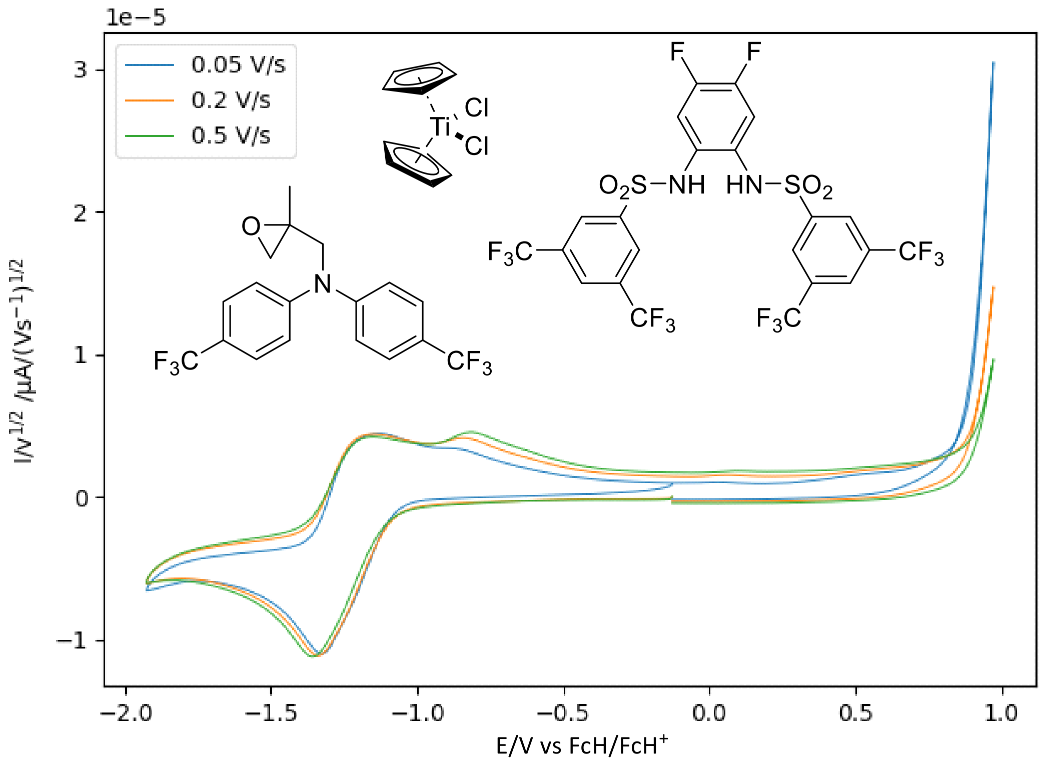


Figure S12: CV of **S5** with $Cp_2TiCl_2$ and **SuA** at different sweep rates.

# 5. Synthesis of the compounds

## 5.1. Substrate synthesis

Bis(4-(trifluoromethyl)phenyl)amine (**A5**)

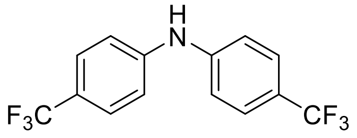


According to a modified literature procedure,[4] in a *Schlenk* flask 1-iodo-4-(trifluoromethyl)benzene (1.00 eq., 20.0 mmol, 5.44 g) is dissolved in dry toluene (54 mL). $Pd(OAc)_2$ (0.15 eq., 3.00 mmol, 0.674 g), $Cs_2CO_3$ (1.50 eq., 30.0 mmol, 9.77 g), XPhos (0.15 eq., 3.00 mmol, 1.43 g) and 4-(trifluoromethyl)aniline (1.00 eq., 20.0 mmol, 3.22 g) are added and the mixture is stirred at 100 °C for 3 h. The suspension is cooled to room temperature, washed once with water (70 mL), once with brine (70 mL) and dried over $MgSO_4$. The solvent is removed under reduced pressure and the crude product is purified by column chromatography (CH:EA:$NEt_3$, 94:5:1 to 90:9:1, $SiO_2$) to obtain **A5** (4.51 g, 14.8 mmol, 74%) as a brown solid.

$^1$H NMR (400 MHz, $CDCl_3$) δ [ppm] 7.59 – 7.51 (m, 4H), 7.16 (d, *J* = 8.5 Hz, 4H), 6.11 (s, 1H).

$^{13}$C NMR (101 MHz, $CDCl_3$) δ [ppm] 144.9, 127.0 (q, *J* = 3.8 Hz), 124.5 (q, *J* = 272.7 Hz), 123.73 (q, *J* = 32.7 Hz), 117.53.

The data is in agreement with literature.[4]

*N*-((2-Methyloxiran-2-yl)methyl)-4-(trifluoromethyl)-*N*-(4-(trifluoromethyl)phenyl)aniline (**S5**)

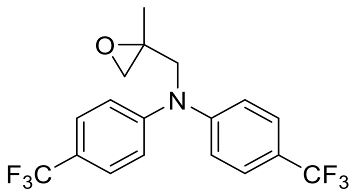


Under *Schlenk* conditions, **A5** (1.00 eq., 10.0 mmol, 3.05 g) and NaH (60% in mineral oils, 1.3 eq., 13.0 mmol, 0.520 g) were dissolved in DMF (25 mL) and stirred for 30 mins. **E1** (2.10 eq., 21.0 mmol, 3.17 g) was added and the mixture was stirred at room temperature overnight. The reaction was quenched by addition of sat. $NH_4Cl$ solution (25 mL) and the aqueous layer was extracted three times with EA (50 mL). The combined organic layers were washed twice with sat. $NH_4Cl$ solution and dried over $MgSO_4$. The crude product was purified by column chromatography (CH:EA:$NEt_3$ 90:9:1, $SiO_2$) to obtain **S5** (2.97 g, 7.91 mmol, 79%) as a colourless solid.

$^1$H NMR (500 MHz, $CDCl_3$) δ [ppm] 7.34 – 7.27 (m, 4H), 6.74 – 6.67 (m, 4H), 3.38 (d, *J* = 16.3 Hz, 1H), 3.22 (d, *J* = 16.3 Hz, 1H), 2.16 (dd, *J* = 4.7, 0.8 Hz, 1H), 2.05 (d, *J* = 4.7 Hz, 1H), 0.90 (d, *J* = 0.7 Hz, 3H).

$^{13}$C NMR (126 MHz, $CDCl_3$) δ [ppm] 150.55, 126.91 (q, *J* = 3.7 Hz), 125.2 (q, *J* = 270.9 Hz) 124.3 (q, *J* = 32.8 Hz), 121.1, 56.30, 55.64, 51.43, 19.39.

IR (neat) [$cm^{-1}$]: 434, 593, 793, 824, 846, 898, 1017, 1061, 1099, 1160, 1248, 1264, 1321, 1517, 1606.

HRMS (EI) calculated for $[M]^{+\bullet}$ 375.1058, found 375.1050.

### 5.2. Titanocene catalyzed radical arylation under electrochemical conditions

2,3-Dihydro-3-(hydroxymethyl)-3-methyl-1-phenylindole **P1**

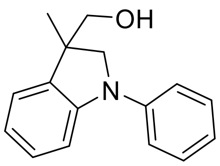


In a divided cell, the anode compartment of the cell was charged with $Cp_2TiCl_2$ (0.28 mmol, 68.2 mg, 0.55 eq.) and $Bu_4NPF_6$ (2.00 eq., 387 mg, 1.00 mmol). The cathode compartment was charged with $Cp_2TiCl_2$ (0.10 eq., 12.5 mg, 0.05 mmol), **SuA** (0.10 eq., 35 mg, 0.05 mmol), $Bu_4NPF_6$ (2.00 eq., 387 mg, 1.00 mmol) and **S1** (1.00 eq., 119 mg, 0.50 mmol). The cell was put inside of a *Glovebox*. THF (5 mL) was added to the cathode compartment and MeCN (5 mL) was added to the anode compartment. The lid including the corresponding electrodes was attached to the cell and the solution was stirred at setting 7 on an IKA RCT basic magnetic stirrer. The electrolysis was conducted at room temperature under constant stirring (immersion depth of the electrodes = 2.6 cm, anode material: GC, cathode material: GC, I = 3 mA, current density = 1.15 mA*$cm^{-2}$, amount of applied charge: 53 C = 1.1 *F*). After completion the cell was removed from the *Glovebox*, the cathode compartment was emptied and rinsed with MTBE and the electrodes were rinsed with MTBE. The conducting salt was filtered off and from the crude $^1$H NMR no formation of the product could be determined.

In a divided cell, the anode compartment of the cell was charged with $Cp_2TiCl_2$ (0.25 mmol, 62.2 mg, 0.50 eq.) and $Bu_4NPF_6$ (2.00 eq., 387 mg, 1.00 mmol). The cathode compartment was charged with $Cp_2TiCl_2$ (0.10 eq., 12.5 mg, 0.05 mmol), **SuA** (0.10 eq., 35 mg, 0.05 mmol), $Bu_4NPF_6$ (2.00 eq., 387 mg, 1.00 mmol) and **S1** (1.00 eq., 119 mg, 0.50 mmol). The cell was put inside of a *Glovebox*. THF (5 mL) was added to the cathode compartment and MeCN (5 mL) was added to the anode compartment. The lid including the corresponding electrodes was attached to the cell and the solution was stirred at setting 7 on an IKA RCT basic magnetic stirrer. The electrolysis was conducted at room temperature under constant stirring (immersion depth of the electrodes = 2.6 cm, anode material: GC, cathode material: GC, I = 3 mA, current density = 1.15 mA*$cm^{-2}$, amount of applied charge: 5 C = 0.1 *F*). After completion the cell was removed from the *Glovebox*, the cathode compartment was emptied and rinsed with MTBE and the electrodes were rinsed with MTBE. The conducting salt was filtered off and from the crude $^1$H NMR 54% formation of the product could be determined.

According to the general procedure, $Cp_2TiCl_2$ (0.10 eq., 12.5 mg, 0.05 mmol), $Bu_4NPF_6$ (2.00 eq., 387 mg, 1.00 mmol) and **S1** (1.00 eq., 120 mg, 0.50 mmol) were reacted (Immersion depth = 3.5 cm, anode material: GC, cathode material: GC, I = 10 mA, current density = 3.57 mA*$cm^{-2}$, charge = 49 C, amount

of applied charge = 1.0 *F*) in THF (5 mL). After workup from the crude $^1H$ NMR no conversion towards the desired product could be observed.

According to the general procedure, $Cp_2TiCl_2$ (0.10 eq., 12.5 mg, 0.05 mmol), **SuA** (0.10 eq., 35 mg, 0.05 mmol), $Bu_4NPF_6$ (2.00 eq., 387 mg, 1.00 mmol) and **S1** (1.00 eq., 120 mg, 0.50 mmol) were reacted (Immersion depth = 3.5 cm, anode material: GC, cathode material: GC I = 10 mA, current density = 3.57 mA*cm$^{-2}$, amount of applied charge: 49 C = 1.0 *F*) in THF (5 mL). After workup from the crude $^1H$ NMR no conversion towards the desired product could be observed and **S1** fully decomposed.

According to the general procedure, $Cp_2TiCl_2$ (0.10 eq., 12.5 mg, 0.05 mmol), **SuA** (0.10 eq., 35 mg, 0.05 mmol), $Bu_4NPF_6$ (2.00 eq., 387 mg, 1.00 mmol) and **S1** (1.00 eq., 120 mg, 0.50 mmol) were reacted (Immersion depth = 3.5 cm, anode material: GC, cathode material: GC, I = 2 mA, current density = 0.71 mA*cm$^{-2}$, amount of applied charge: 49 C = 1.0 *F*) in THF (5 mL). After workup from the crude $^1H$ NMR a conversion of 87% towards **P1** could be observed.

According to the general procedure, $Cp_2TiCl_2$ (0.10 eq., 12.5 mg, 0.05 mmol), **SuA** (0.10 eq., 35 mg, 0.05 mmol), $Bu_4NPF_6$ (2.00 eq., 387 mg, 1.00 mmol) and **S1** (1.00 eq., 120 mg, 0.50 mmol) were reacted (Immersion depth = 4.0 cm, anode material: GC, cathode material: GC, I = 3 mA, current density = 0.94 mA*cm$^{-2}$, amount of applied charge: 53 C = 1.1 *F*) in THF (5 mL). From the crude $^1H$ NMR spectrum a NMR yield of 90% against $CH_2Br_2$ could be determined. After workup the crude product was purified by automated flash column chromatography to obtain **P1** as a colourless solid (102 mg, 0.43 mmol, 85%).

According to the general procedure, $Cp_2TiCl_2$ (0.10 eq., 12.5 mg, 0.05 mmol), $Bu_4NPF_6$ (2.00 eq., 387 mg, 1.00 mmol) and **S1** (1.00 eq., 120 mg, 0.50 mmol) were reacted (Immersion depth = 4.0 cm, anode material: GC, cathode material: GC, I = 3 mA, current density = 0.94 mA*cm$^{-2}$, charge = 53 C, amount of applied charge = 1.1 *F*) in THF (5 mL). From the crude $^1H$ NMR a NMR yield of 70% against $CH_2Br_2$ could be determined.

Under *Schlenk* conditions, $Cp_2TiCl_2$ (0.10 eq., 12.5 mg, 0.05 mmol), **SuA** (0.10 eq., 35 mg, 0.05 mmol), $Bu_4NPF_6$ (2.00 eq., 387 mg, 1.00 mmol) and **S1** (1.00 eq., 120 mg, 0.50 mmol) were added to freshly distilled THF (5 mL) and stirred for 6 h at room temperature. The mixture was diluted with $Et_2O$ and the conducting salt was filtered off. After workup from the crude $^1H$ NMR no conversion towards the desired product could be observed.

According to the general procedure, **SuA** (0.10 eq., 35 mg, 0.05 mmol), $Bu_4NPF_6$ (2.00 eq., 387 mg, 1.00 mmol) and **S1** (1.00 eq., 120 mg, 0.50 mmol) were reacted (Immersion depth = 4.0 cm, anode material: GC, cathode material: GC, I = 3 mA, current density = 0.94 mA*cm$^{-2}$, amount of applied charge: 53

C = 1.1 *F*) in THF (5 mL). After workup from the crude $^1$H NMR no conversion towards the desired product could be observed.

$^1$H NMR (400 MHz, $C_6D_6$) δ 7.23 – 7.17 (m, 2H), 7.13 – 7.09 (m, 2H), 7.03 (td, *J* = 7.7, 1.4 Hz, 1H), 6.93 (dd, *J* = 7.3, 1.4 Hz, 1H), 6.91 – 6.85 (m, 1H), 6.76 (td, *J* = 7.4, 1.0 Hz, 1H), 3.61 (d, *J* = 9.4 Hz, 1H), 3.32 – 3.24 (m, 2H), 3.20 (d, *J* = 10.5 Hz, 1H), 1.14 (s, 3H).

$^{13}$C NMR (126 MHz, $C_6D_6$) δ 147.2, 144.6, 136.4, 129.6, 128.5, 123.8, 121.4, 119.5, 118.1, 109.0, 69.0, 61.9, 45.7, 22.2

The data is in agreement with literature.[2]

5-Bromo-1-(4-bromophenyl)-2,3-dihydro-3-(hydroxymethyl)-3-methylindole **P2**

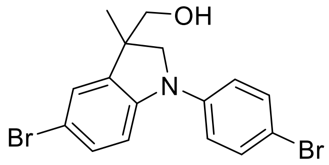


In a divided cell, the anode compartment of the cell was charged with $Cp_2TiCl_2$ (0.25 mmol, 62.2 mg, 0.50 eq.) and $Bu_4NPF_6$ (2.00 eq., 387 mg, 1.00 mmol). The cathode compartment was charged with $Cp_2TiCl_2$ (0.10 eq., 12.5 mg, 0.05 mmol), **SuA** (0.10 eq., 35 mg, 0.05 mmol), $Bu_4NPF_6$ (2.00 eq., 387 mg, 1.00 mmol) and **S2** (1.00 eq., 198 mg, 0.50 mmol). The cell was put inside of a *Glovebox*. THF (5 mL) was added to the cathode compartment and MeCN (5 mL) was added to the anode compartment. The lid including the corresponding electrodes was attached to the cell and the solution was stirred at setting 7 on an IKA RCT basic magnetic stirrer. The electrolysis was conducted at room temperature under constant stirring (immersion depth of the electrodes = 2.6 cm, anode material: GC, cathode material: GC, I = 3 mA, current density = 1.15 mA*cm$^{-2}$, amount of applied charge: 5 C = 0.1 *F*). After completion the cell was removed from the *Glovebox*, the cathode compartment was emptied and rinsed with MTBE and the electrodes were rinsed with MTBE. The conducting salt was filtered off and from the crude $^1$H NMR no formation of the product could be determined.

In a divided cell, the anode compartment of the cell was charged with $Cp_2TiCl_2$ (0.63 mmol, 155 mg, 1.25 eq.) and $Bu_4NPF_6$ (2.00 eq., 387 mg, 1.00 mmol). The cathode compartment was charged with $Cp_2TiCl_2$ (0.10 eq., 12.5 mg, 0.05 mmol), **SuA** (0.10 eq., 35 mg, 0.05 mmol), $Bu_4NPF_6$ (2.00 eq., 387 mg, 1.00 mmol) and **S2** (1.00 eq., 198 mg, 0.50 mmol). The cell was put inside of a *Glovebox*. THF (5 mL) was added to the cathode compartment and MeCN (5 mL) was added to the anode compartment. The lid including the corresponding electrodes was attached to the cell and the solution was stirred at setting 7 on an IKA RCT basic magnetic stirrer. The electrolysis was conducted at room temperature under constant stirring (immersion depth of the electrodes = 2.6 cm, anode material: GC, cathode

material: GC, I = 3 mA, current density = 1.15 mA*cm$^{-2}$, amount of applied charge: 5 C = 0.1 *F*). After completion the cell was removed from the *Glovebox*, the cathode compartment was emptied and rinsed with MTBE and the electrodes were rinsed with MTBE. The conducting salt was filtered off and from the crude $^1$H NMR no formation of the product could be determined.

According to the general procedure, $Cp_2TiCl_2$ (0.10 eq., 12.5 mg, 0.05 mmol), **SuA** (0.10 eq., 35 mg, 0.05 mmol), $Bu_4NPF_6$ (2.00 eq., 387 mg, 1.00 mmol) and **S2** (1.00 eq., 199 mg, 0.50 mmol) were reacted (Immersion depth = 3.5 cm, anode material: GC, cathode material: GC, I = 2 mA, current density = 0.71 mA*cm$^{-2}$, amount of applied charge: 49 C = 1.0 *F*) in THF (5 mL). After workup from the crude $^1$H NMR a conversion of 25% towards **P2** could be observed.

According to the general procedure, $Cp_2TiCl_2$ (0.10 eq., 12.5 mg, 0.05 mmol), **SuA** (0.10 eq., 35 mg, 0.05 mmol), $Bu_4NPF_6$ (2.00 eq., 387 mg, 1.00 mmol) and **S2** (1.00 eq., 199 mg, 0.50 mmol) were reacted (Immersion depth = 3.5 cm, anode material: RVC 100 ppi, cathode material: GC, I = 2 mA, current density = 0.71 mA*cm$^{-2}$, amount of applied charge: 49 C = 1.0 *F*) in THF (5 mL). After workup from the crude $^1$H NMR full decomposition of the starting material could be observed.

According to the general procedure, $Cp_2TiCl_2$ (0.10 eq., 12.5 mg, 0.05 mmol), **SuA** (0.10 eq., 35 mg, 0.05 mmol), $Bu_4NPF_6$ (2.00 eq., 387 mg, 1.00 mmol) and **S2** (1.00 eq., 199 mg, 0.50 mmol) were reacted (Immersion depth = 3.5 cm, anode material: Pt, cathode material: GC, I = 2 mA, current density = 0.71 mA*cm$^{-2}$, amount of applied charge: 49 C = 1.0 *F*) in THF (5 mL). After workup from the crude $^1$H NMR full decomposition of the starting material could be observed.

According to the general procedure, $Cp_2TiCl_2$ (0.10 eq., 12.5 mg, 0.05 mmol), **SuA** (0.10 eq., 35 mg, 0.05 mmol), $Bu_4NPF_6$ (2.00 eq., 387 mg, 1.00 mmol) and **S2** (1.00 eq., 199 mg, 0.50 mmol) were reacted (Immersion depth = 4.0 cm, anode material: GC, cathode material: GC, I = 3 mA, current density = 0.94 mA*cm$^{-2}$, amount of applied 121 C = 2.5 *F*) in THF (5 mL). After workup the crude product was purified by automated flash column chromatography to obtain **P2** as a colourless solid (161 mg, 0.41 mmol, 81%).

$^1$H NMR (400 MHz, $C_6D_6$) δ 7.28 – 7.21 (m, 2H), 7.14 – 7.09 (m, 2H), 6.66 – 6.54 (m, 3H), 3.34 (d, *J* = 9.4 Hz, 1H), 3.05 (d, *J* = 10.5 Hz, 1H), 2.96 (dd, *J* = 10.2, 8.0 Hz, 2H), 0.92 (s, 3H).

$^{13}$C NMR (176 MHz, $C_6D_6$) δ 145.6, 142.9, 139.2, 132.6, 131.1, 127.1, 119.5, 113.8, 111.5, 110.3, 68.6, 61.9, 45.6, 22.1.

The data is in agreement with literature.[2]

2,3-Dihydro-3,5-dimethyl-3-hydroxymethyl-1-(4-methylphenyl)indole **P3**

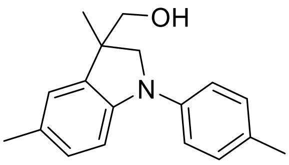

According to the general procedure, $Cp_2TiCl_2$ (0.10 eq., 12.5 mg, 0.05 mmol), **SuA** (0.10 eq., 35 mg, 0.05 mmol), $Bu_4NPF_6$ (2.00 eq., 387 mg, 1.00 mmol) and **S3** (1.00 eq., 133 mg, 0.50 mmol) were reacted (Immersion depth = 4.0 cm, anode material: GC, cathode material: GC, I = 3 mA, current density = 0.94 mA*cm$^{-2}$, amount of applied charge 121 C = 2.5 *F*) in THF (5 mL). After workup the crude product was purified by automated flash column chromatography to obtain **P3** as a colourless oil (114 mg, 0.43 mmol, 86%).

$^1$H NMR (400 MHz, $C_6D_6$) δ 7.15 – 7.09 (m, 3H), 7.04 (d, *J* = 8.4 Hz, 2H), 6.88 – 6.82 (m, 2H), 3.66 (d, *J* = 9.3 Hz, 1H), 3.38 – 3.31 (m, 2H), 3.27 (d, *J* = 10.6 Hz, 1H), 2.20 (d, *J* = 1.6 Hz, 6H).

$^{13}$C NMR (126 MHz, $C_6D_6$) δ 145.4, 142.5, 136.3, 130.1, 130.0, 128.6, 124.3, 118.0, 108.6, 68.9, 62.1, 45.6, 22.1, 20.9, 20.8.

The data is in agreement with literature.[2]

1-(4-Fluorophenyl)- 5-fluoro-3-(hydroxymethyl)-3-methyl-2,3-dihydroindole **P4**

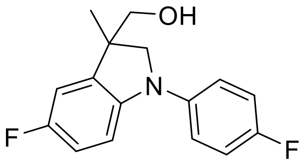

According to the general procedure, $Cp_2TiCl_2$ (0.10 eq., 12.5 mg, 0.05 mmol), **SuA** (0.10 eq., 35 mg, 0.05 mmol), $Bu_4NPF_6$ (2.00 eq., 387 mg, 1.00 mmol) and **S4** (1.00 eq., 137 mg, 0.50 mmol) were reacted (Immersion depth = 4.0 cm, anode material: GC, cathode material: GC, I = 3 mA, current density = 0.94 mA*cm$^{-2}$, amount of applied charge: 121 C = 2.5 *F*) in THF (5 mL). After workup the crude product was purified by automated flash column chromatography to obtain **P4** as a colourless solid (104 mg, 0.38 mmol, 76%).

$^1$H NMR (400 MHz, $C_6D_6$) δ 6.88 – 6.80 (m, 2H), 6.77 – 6.61 (m, 5H), 3.43 (d, *J* = 9.3 Hz, 1H), 3.16 (d, *J* = 10.6 Hz, 1H), 3.08 (dd, *J* = 13.1, 9.9 Hz, 2H), 1.01 (s, 3H).

$^{13}$C NMR (126 MHz, $C_6D_6$) δ 159.0 (d, *J* = 88.6 Hz), 157.1 (d, *J* = 87.9), 143.82 (d, *J* = 1.3 Hz), 141.0 (d, *J* = 2.5 Hz), 138.2 (d, *J* = 7.6 Hz), 119.9 (d, *J* = 7.6 Hz), 116.3 (d, *J* = 22.7 Hz), 114.3 (d, *J* = 23.9 Hz), 111.7 (d *J* = 24.0 Hz), 108.8 (d, *J* = 8.8 Hz), 68.68, 62.85, 45.8 (d, *J* = 1.3 Hz), 22.08.

The data is in agreement with literature.[2]

2,3-Dihydro-3-hydroxymethyl-3-methyl 5-trifluoromethyl-1-(4-(trifluoromethyl)phenyl)indole **P5**

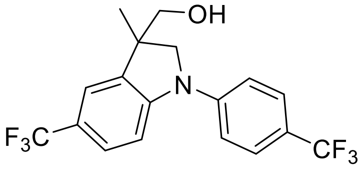


In a divided cell, the anode compartment of the cell was charged with $Cp_2TiCl_2$ (0.25 mmol, 62.2 mg, 0.50 eq.) and $Bu_4NPF_6$ (2.00 eq., 387 mg, 1.00 mmol). The cathode compartment was charged with $Cp_2TiCl_2$ (0.10 eq., 12.5 mg, 0.05 mmol), **SuA** (0.10 eq., 35 mg, 0.05 mmol), $Bu_4NPF_6$ (2.00 eq., 387 mg, 1.00 mmol) and **S5** (1.00 eq., 187 mg, 0.50 mmol). The cell was put inside of a *Glovebox*. THF (5 mL) was added to the cathode compartment and MeCN (5 mL) was added to the anode compartment. The lid including the corresponding electrodes was attached to the cell and the solution was stirred at setting 7 on an IKA RCT basic magnetic stirrer. The electrolysis was conducted at room temperature under constant stirring (immersion depth of the electrodes = 2.6 cm, anode material: GC, cathode material: GC, I = 3 mA, current density = 1.15 $mA*cm^{-2}$, amount of applied charge: 49 C = 1.0 *F*). After completion the cell was removed from the *Glovebox*, the cathode compartment was emptied and rinsed with MTBE and the electrodes were rinsed with MTBE. The conducting salt was filtered off and from the crude $^1H$ NMR no formation of the product could be determined.

In a divided cell, the anode compartment of the cell was charged with $Cp_2TiCl_2$ (0.25 mmol, 62.2 mg, 0.50 eq.) and $Bu_4NPF_6$ (2.00 eq., 387 mg, 1.00 mmol). The cathode compartment was charged with $Cp_2TiCl_2$ (0.10 eq., 12.5 mg, 0.05 mmol), **SuA** (0.10 eq., 35 mg, 0.05 mmol), $Bu_4NPF_6$ (2.00 eq., 387 mg, 1.00 mmol) and **S5** (1.00 eq., 187 mg, 0.50 mmol). The cell was put inside of a *Glovebox*. THF (5 mL) was added to the cathode compartment and MeCN (5 mL) was added to the anode compartment. The lid including the corresponding electrodes was attached to the cell and the solution was stirred at setting 7 on an IKA RCT basic magnetic stirrer. The electrolysis was conducted at room temperature under constant stirring (immersion depth of the electrodes = 2.6 cm, anode material: GC, cathode material: GC, I = 3 mA, current density = 1.15 $mA*cm^{-2}$, amount of applied charge 5 C = 0.1 *F*). Afterwards, the reaction was stirred at room temperature for 4h without current. After completion the cell was removed from the *Glovebox*, the cathode compartment was emptied and rinsed with MTBE and the electrodes were rinsed with MTBE. The conducting salt was filtered off and from the crude $^1H$ NMR no formation of the product could be determined.

According to the general procedure, $Cp_2TiCl_2$ (0.10 eq., 12.5 mg, 0.05 mmol), **SuA** (0.10 eq., 35 mg, 0.05 mmol), $Bu_4NPF_6$ (2.00 eq., 387 mg, 1.00 mmol) and **S5** (1.00 eq., 187 mg, 0.50 mmol) were reacted (Immersion depth = 4.0 cm, anode material: GC, cathode material: GC, I = 3 mA, current density = 0.94 $mA*cm^{-2}$, amount of applied charge: 49 C = 1.0 *F*) in THF (5 mL). After workup the crude product

was purified by automated flash column chromatography to obtain **P5** as a colourless solid (114 mg, 0.31 mmol, 61%).

$^1$H NMR (499 MHz, $C_6D_6$) δ 7.41 – 7.35 (m, 2H), 7.32 (d, *J* = 1.9 Hz, 1H), 7.29 (ddd, *J* = 8.4, 2.0, 0.9 Hz, 1H), 6.77 (d, *J* = 8.4 Hz, 1H), 6.75 – 6.70 (m, 2H), 3.42 (d, *J* = 9.5 Hz, 1H), 3.03 (dd, *J* = 10.0, 4.9 Hz, 2H), 2.94 (d, *J* = 10.5 Hz, 1H), 0.93 (s, 3H).

$^{13}$C NMR (126 MHz, $C_6D_6$) δ 148.4, 145.8, 137.6, 126.8 (q, *J* = 3.7 Hz), 126.3 (q, *J* = 4.0 Hz), 124.4 (q, *J* = 46.5 Hz), 123.3 (q, *J* = 32.7 Hz), 122.5 – 121.7 (m), 120.9 (q, *J* = 3.6 Hz), 117.2, 108.6, 68.3, 61.6, 45.1, 21.8.

IR (neat) [cm$^{-1}$]: 585, 615, 814, 901, 106, 1099, 1160, 1249, 1266, 1312, 1495, 1525, 1608, 2868, 2934, 3347.

HRMS (EI) calculated $[M]^{+\bullet}$ 375.1052, found 375.1046.

2,3-Dihydro-1,3-dimethyl-3-hydroxymethyl-5-(trifluoromethyl)indole **P6**

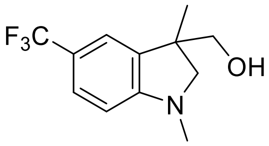


According to the general procedure, $Cp_2TiCl_2$ (0.10 eq., 12.5 mg, 0.05 mmol), **SuA** (0.10 eq., 35 mg, 0.05 mmol), $Bu_4NPF_6$ (2.00 eq., 387 mg, 1.00 mmol) and **S6** (1.00 eq., 122 mg, 0.50 mmol) were reacted (Immersion depth = 4.0 cm, anode material: GC, cathode material: GC, I = 3 mA, current density = 0.94 mA*cm$^{-2}$, amount of applied charge: 74 C = 1.5 *F*) in THF (5 mL). After workup the crude product was purified by automated flash column chromatography to obtain **P6** as a colourless solid (62 mg, 0.25 mmol, 50%).

$^1$H NMR (400 MHz, $C_6D_6$) δ 7.37 (ddd, *J* = 8.2, 1.9, 0.9 Hz, 1H), 7.27 (d, *J* = 1.9 Hz, 1H), 6.00 (d, *J* = 8.2 Hz, 1H), 3.16 (d, *J* = 10.4 Hz, 1H), 3.07 (dd, *J* = 10.4, 9.0 Hz, 2H), 2.57 (d, *J* = 9.0 Hz, 1H), 2.22 (s, 3H), 0.98 (s, 3H).

$^{13}$C NMR (101 MHz, $C_6D_6$) δ 155.4, 135.4, 126.6 (q, *J* = 4.1 Hz), 120.0 (q, *J* = 3.6 Hz), 119.2 (q, *J* = 31.9 Hz), 105.9, 68.3, 64.7, 45.8, 34.1, 21.9.

The data is in agreement with literature.[2]

5-Bromo-2,3-dihydro-1,3-dimethyl-3-(hydroxymethyl)indole **P7**

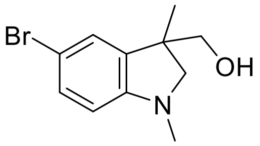


According to the general procedure, $Cp_2TiCl_2$ (0.10 eq., 12.5 mg, 0.05 mmol), **SuA** (0.10 eq., 35 mg, 0.05 mmol), $Bu_4NPF_6$ (2.00 eq., 387 mg, 1.00 mmol) and **S6** (1.00 eq., 128 mg, 0.50 mmol) were reacted (Immersion depth = 4.0 cm, anode material: GC, cathode material: GC, I = 3 mA, current density = 0.94 mA*cm$^{-2}$, amount of applied charge: 172 C = 3.5 *F*) in THF (5 mL). After workup the crude product was purified by automated flash column chromatography to obtain **P6** as a colourless solid (82 mg, 0.32 mmol, 64%).

$^{1}$H NMR (400 MHz, $C_6D_6$) δ 7.21 (dd, *J* = 8.3, 2.1 Hz, 1H), 7.12 (d, *J* = 2.0 Hz, 1H), 5.98 (d, *J* = 8.3 Hz, 1H), 3.22 (d, *J* = 10.5 Hz, 1H), 3.12 (d, *J* = 10.5 Hz, 1H), 3.07 (d, *J* = 8.8 Hz, 1H), 2.55 (d, *J* = 8.8 Hz, 1H), 2.25 (s, 3H), 1.01 (s, 3H).

$^{13}$C NMR (126 MHz, $C_6D_6$) δ 152.3, 137.6, 131.2, 126.3, 109.8, 108.8, 68.4, 65.3, 46.2, 35.1, 21.8.

The data is in agreement with literature.[2]

## 6. Spectra

$^{1}$H NMR of **S5**

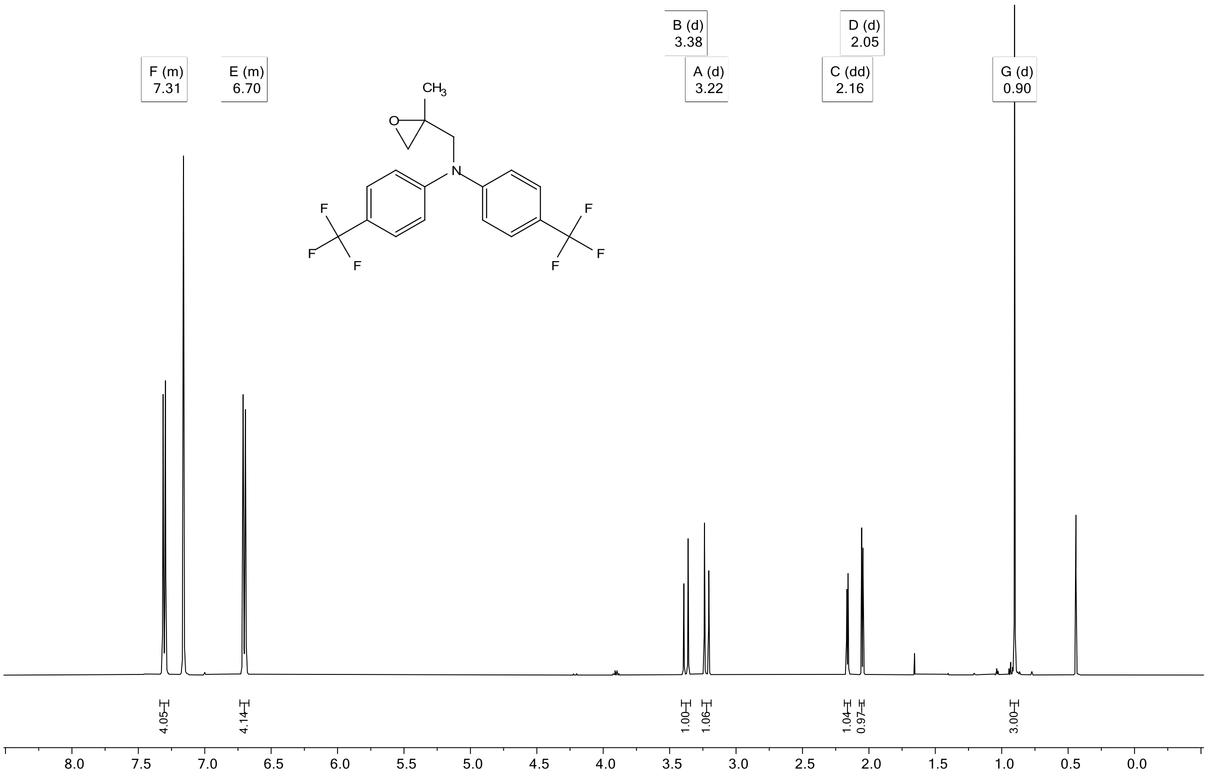

$^{13}C$ NMR of **S5**

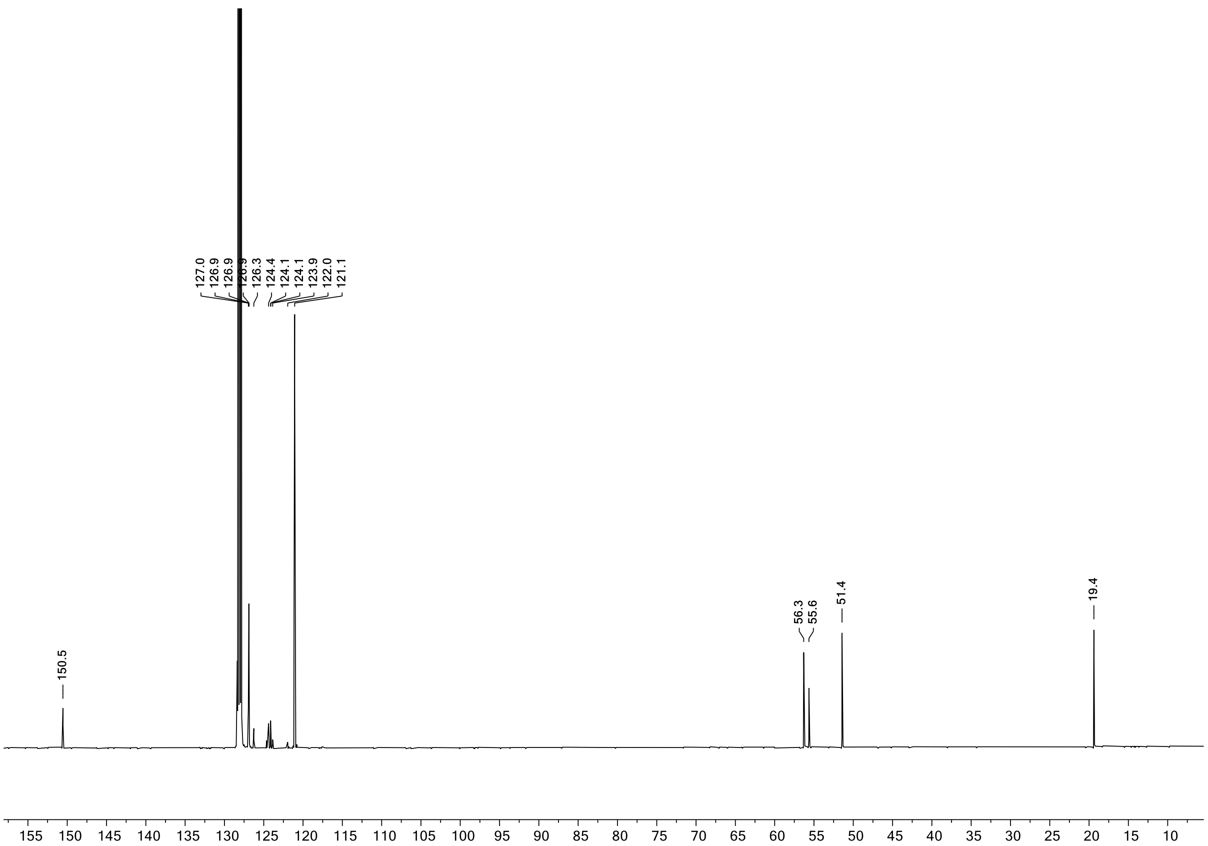


$^{1}H$ NMR of **P1**

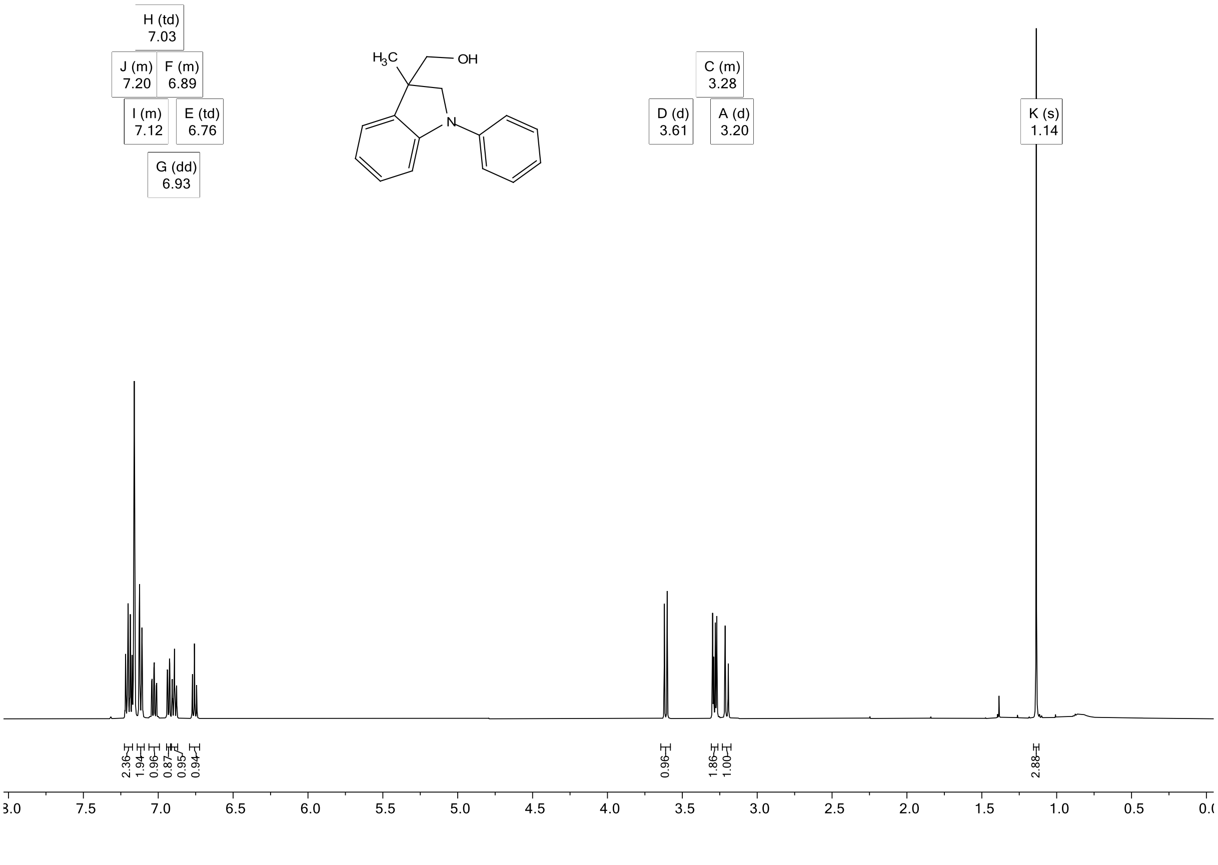

$^{13}$C NMR of **P1**

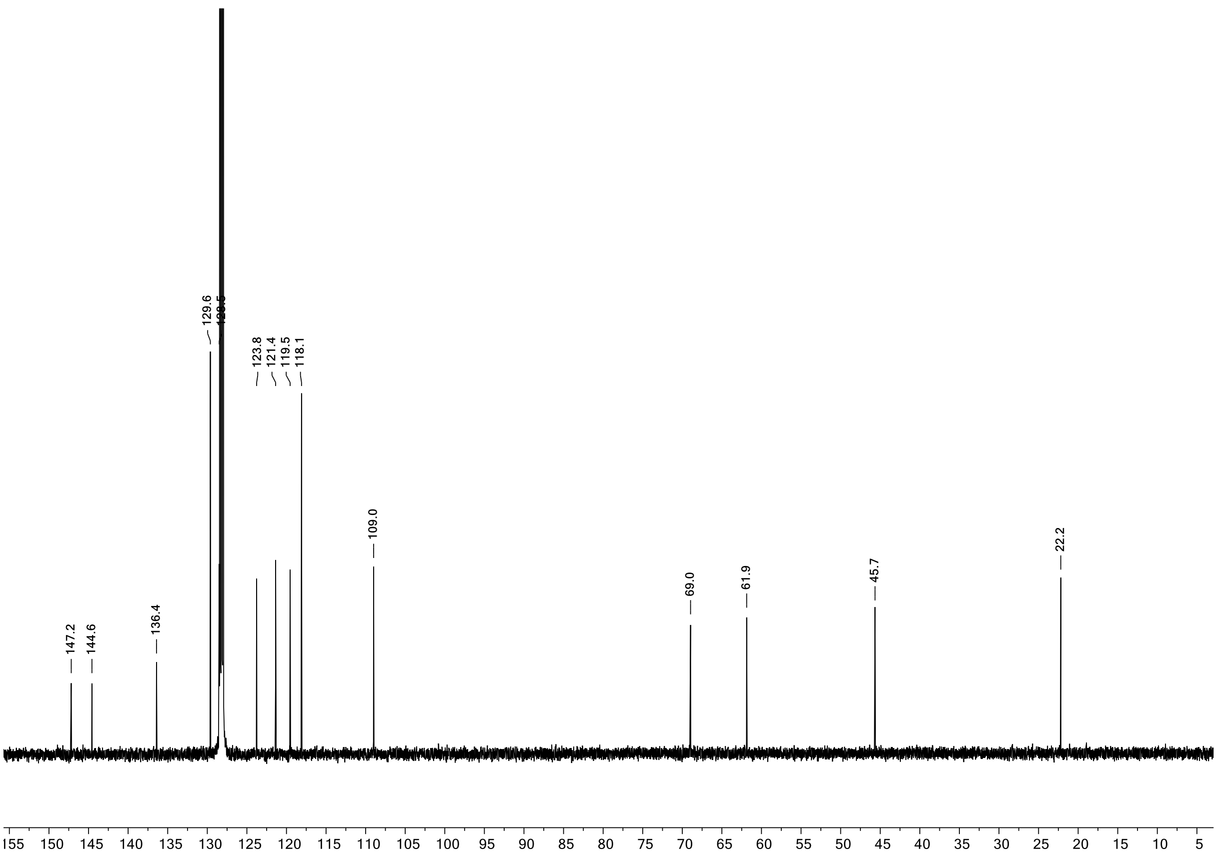


$^{1}$H NMR of **P2**

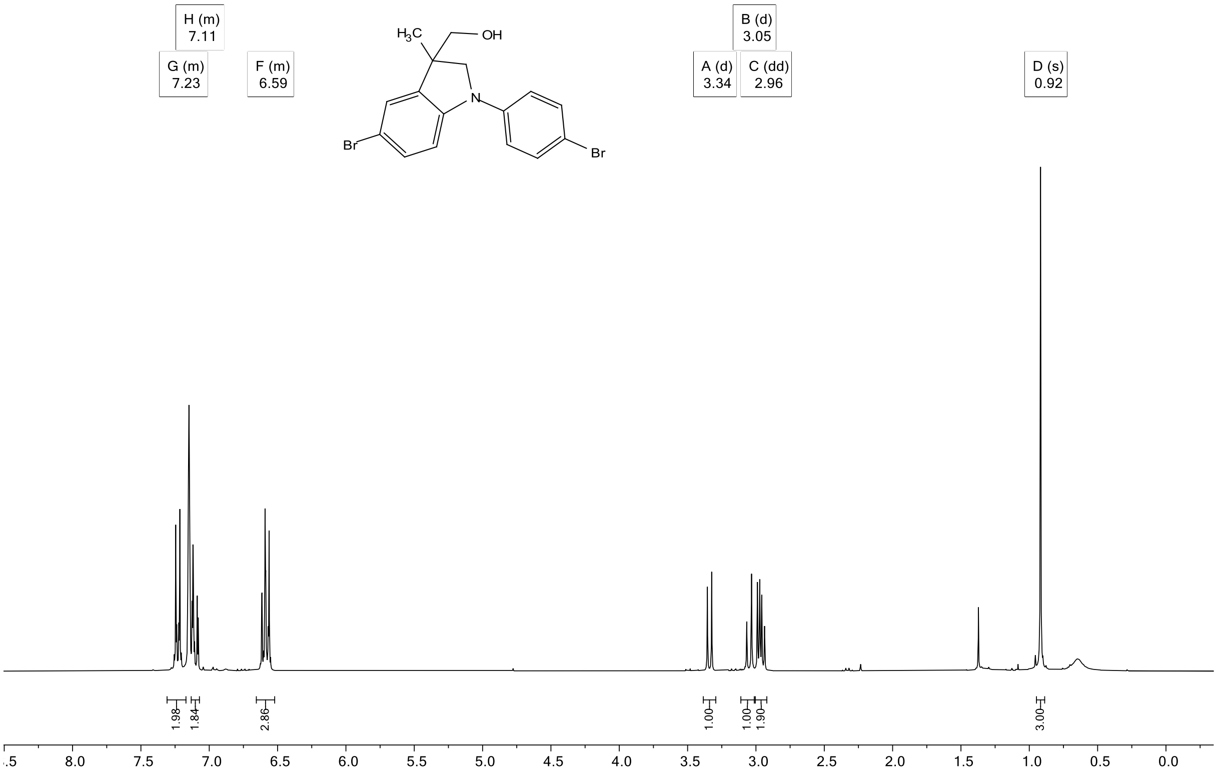

$^{13}C$ NMR of **P2**

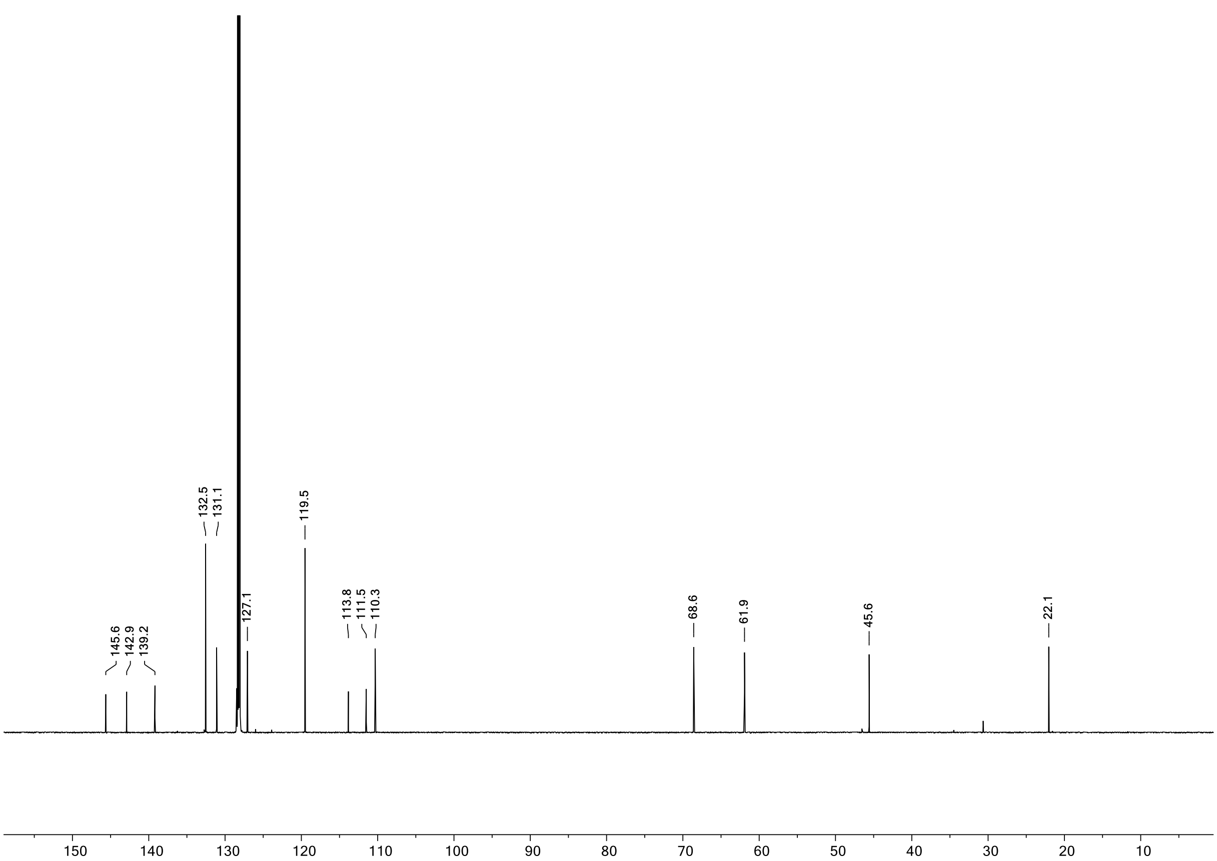


$^{1}H$ NMR of **P3**

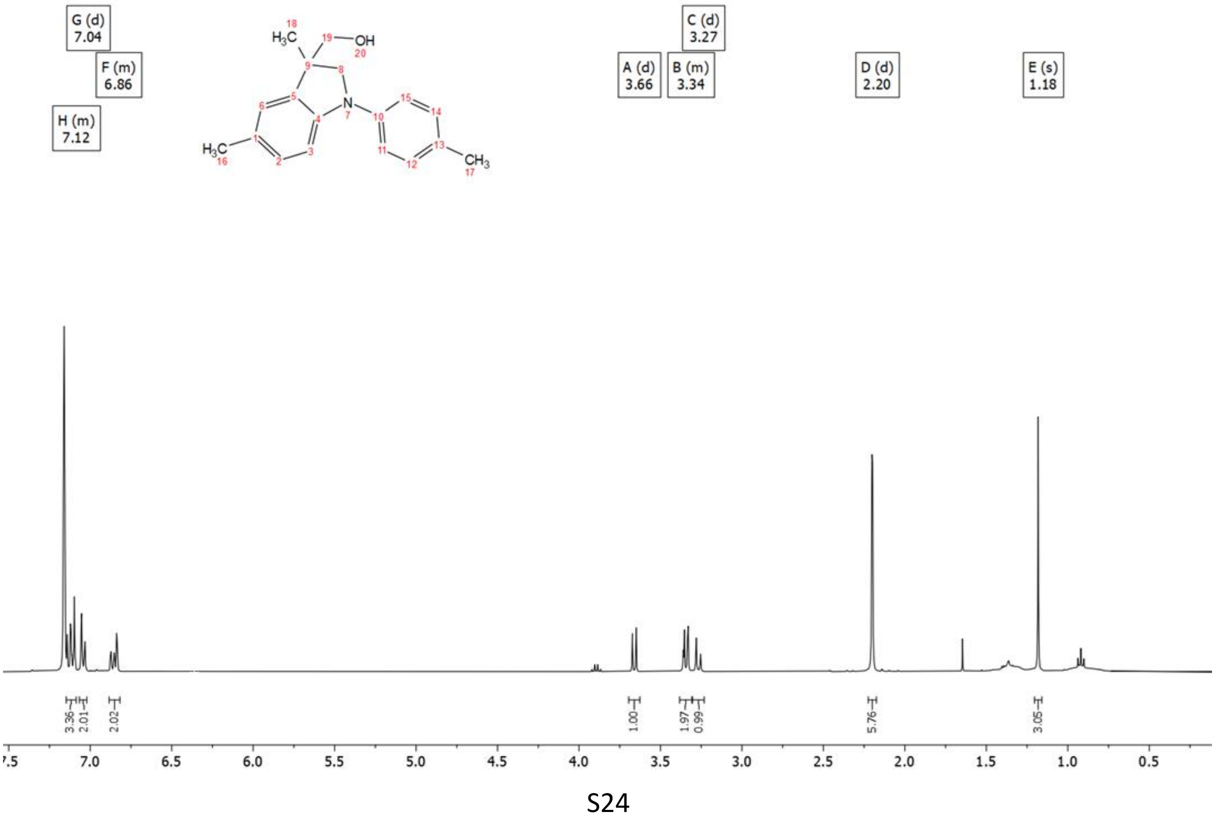

$^{13}C$ NMR of **P3**

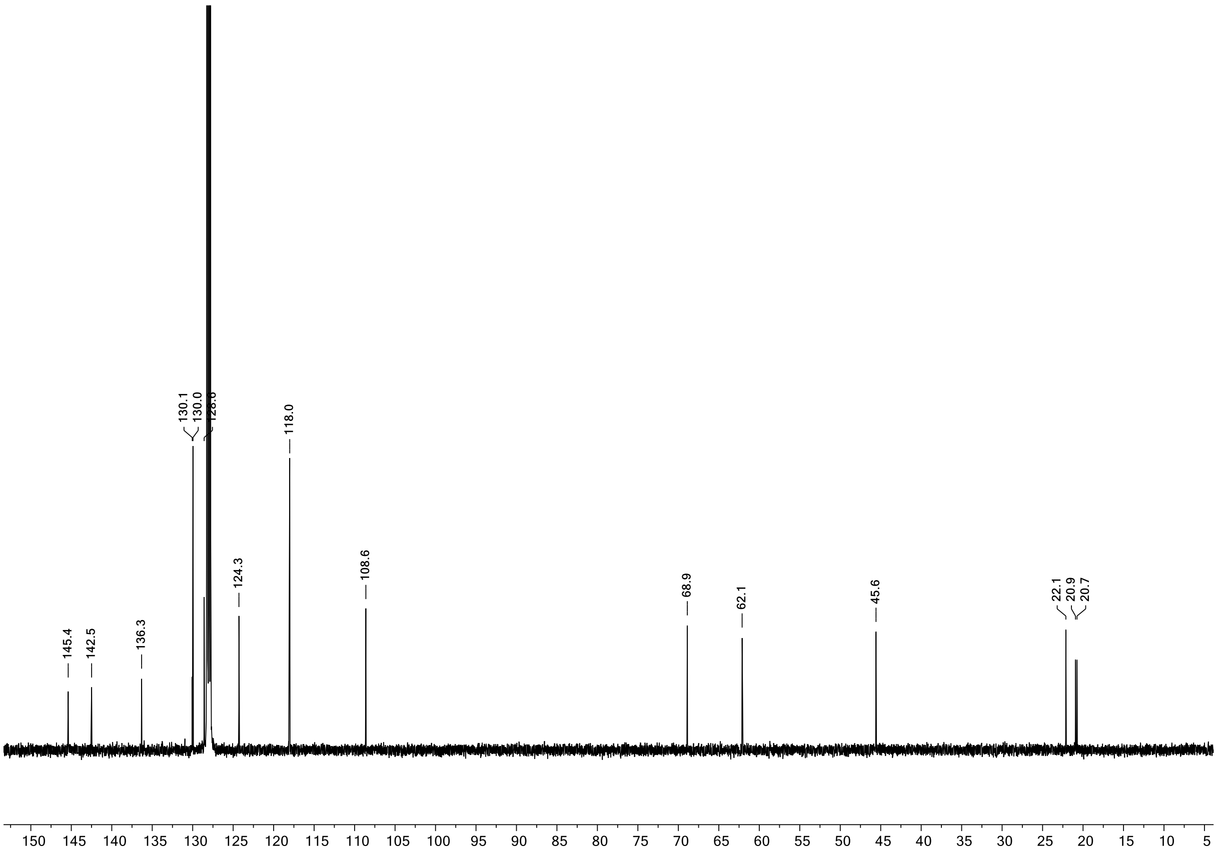


$^{1}H$ NMR of **P4**

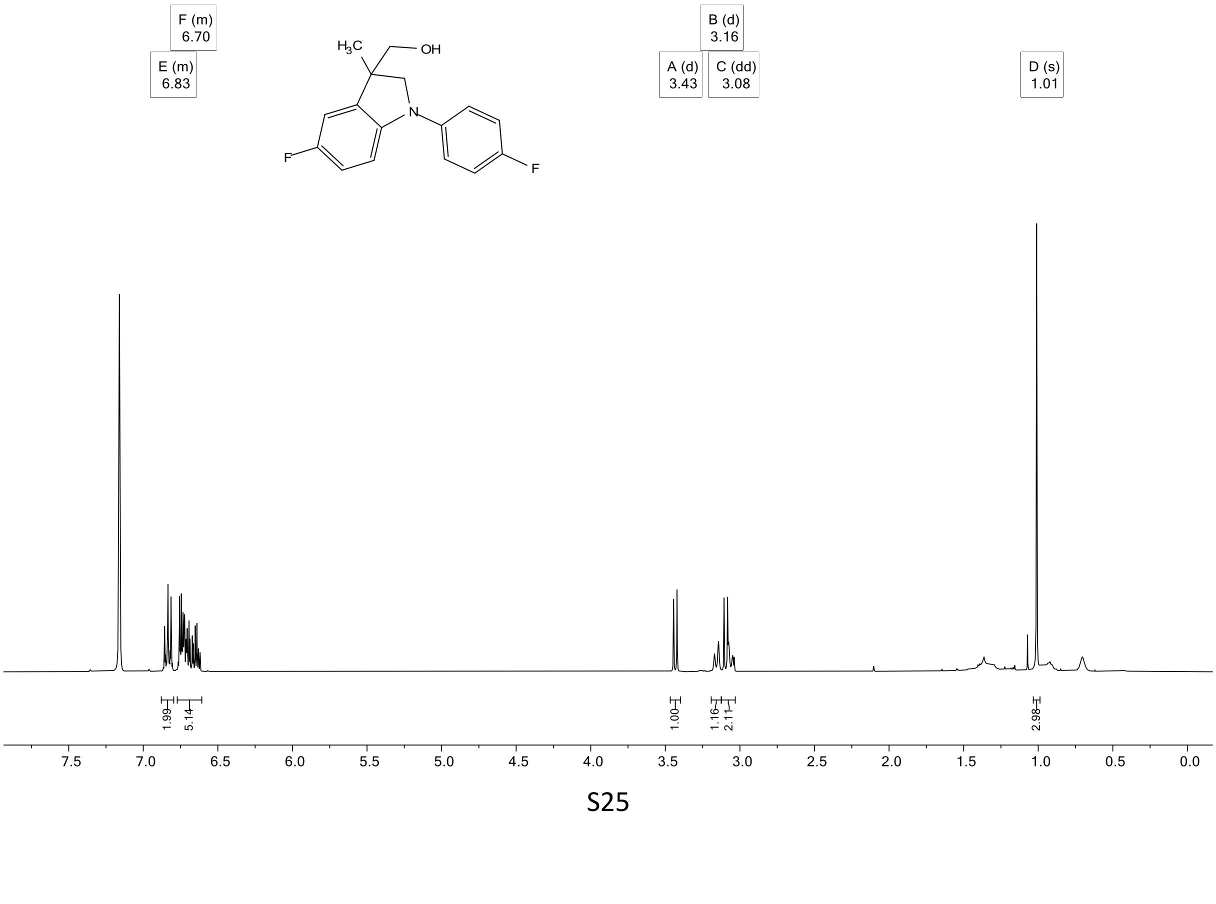

$^{13}$C NMR of **P4**

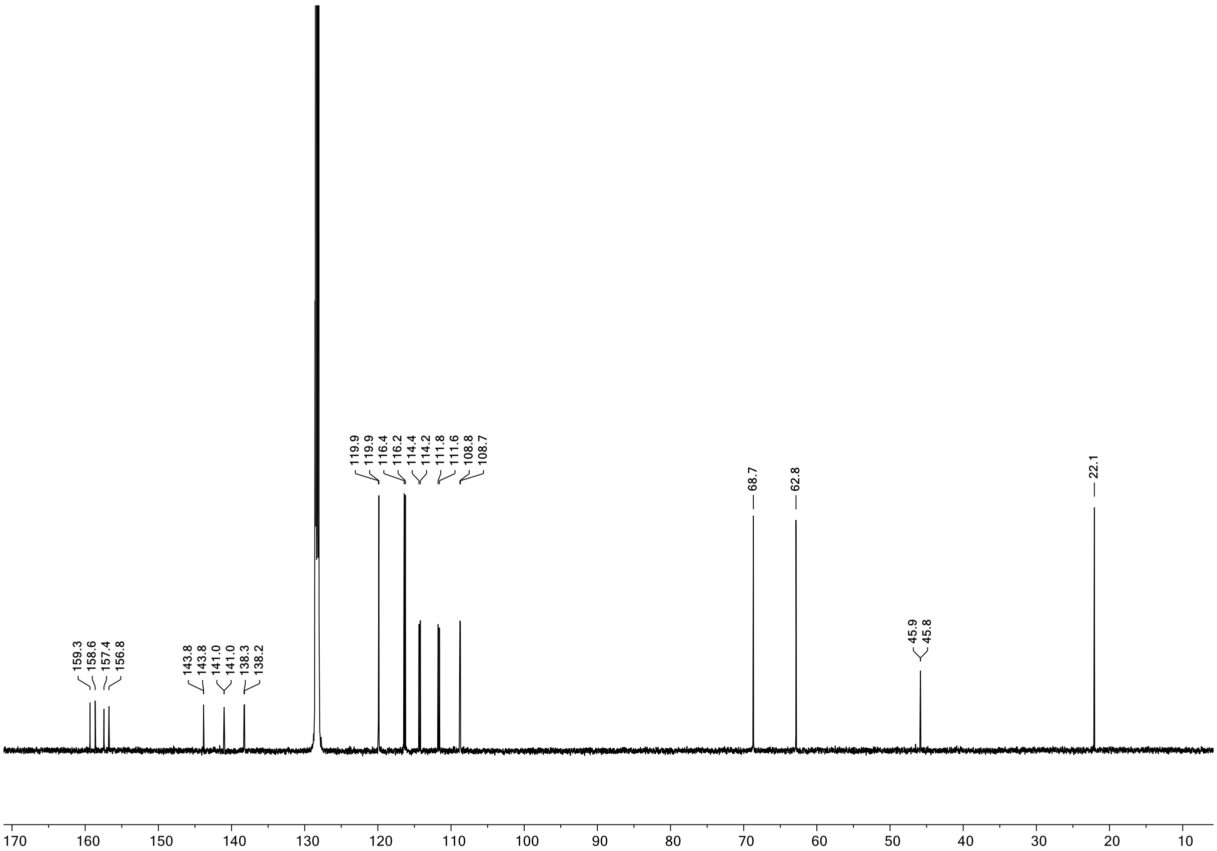


$^{1}$H NMR of **P5**

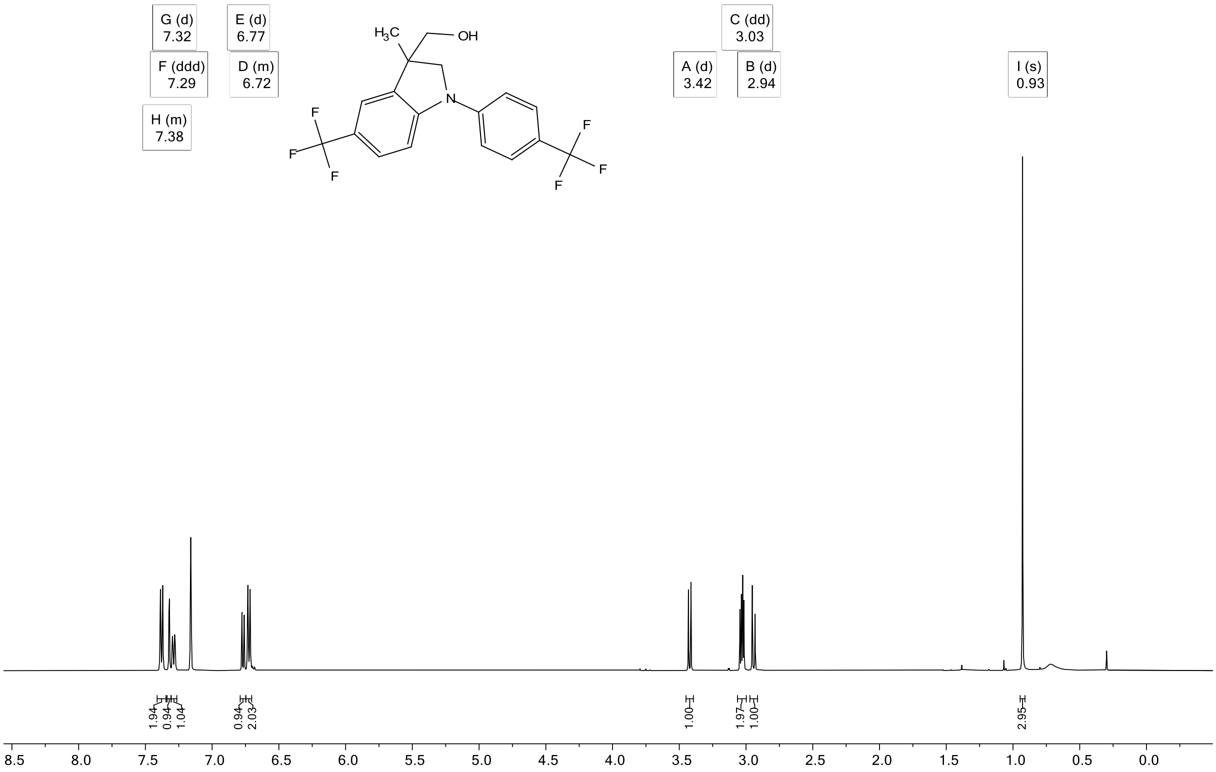

$^{13}$C NMR of **P5**

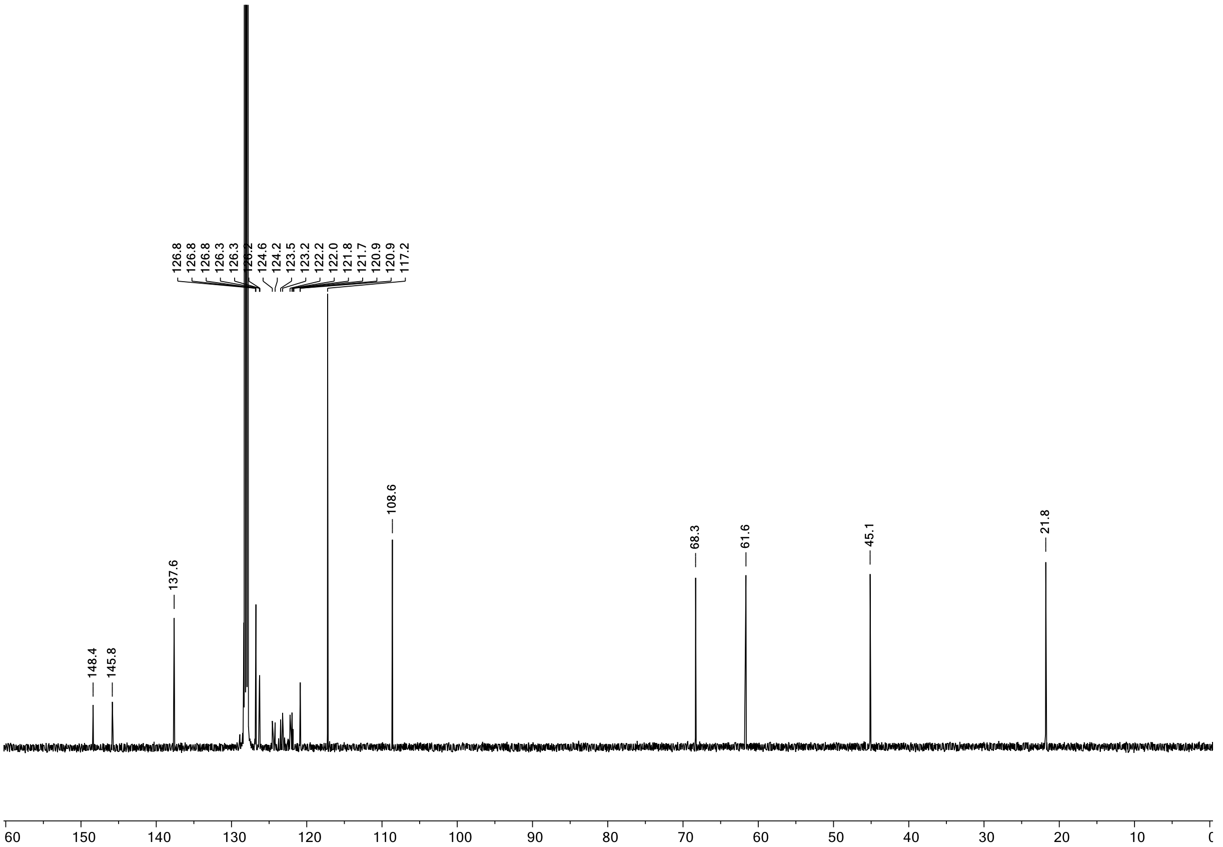


$^{1}$H NMR of **P6**

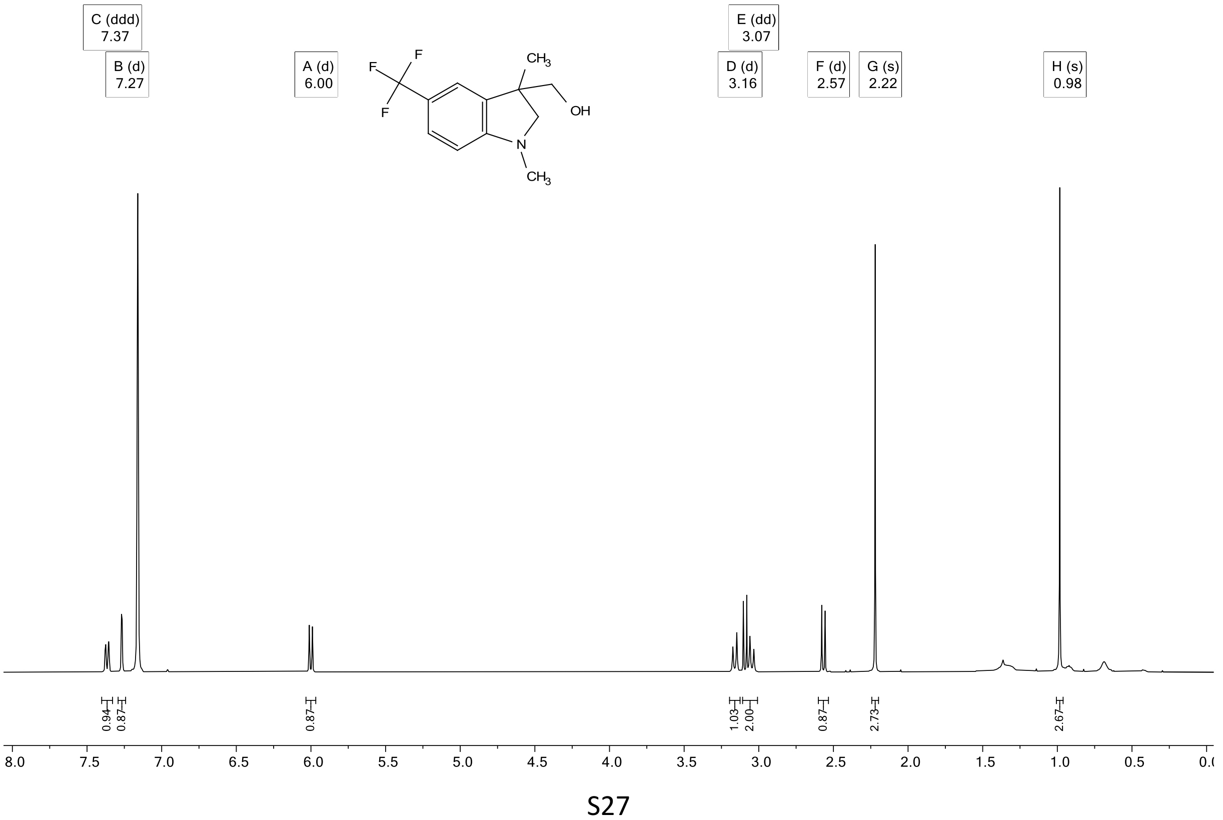

$^{13}C$ NMR of **P6**

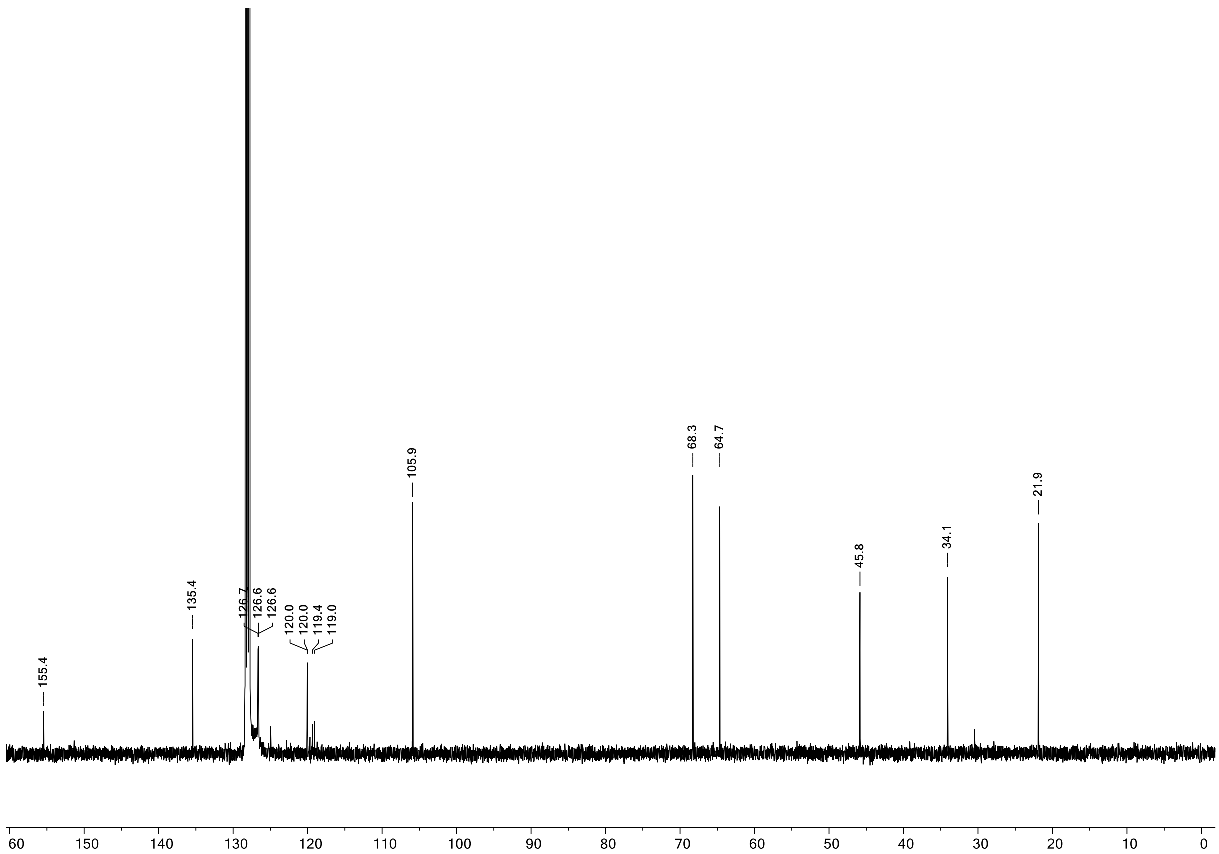


$^{1}H$ NMR of **P7**

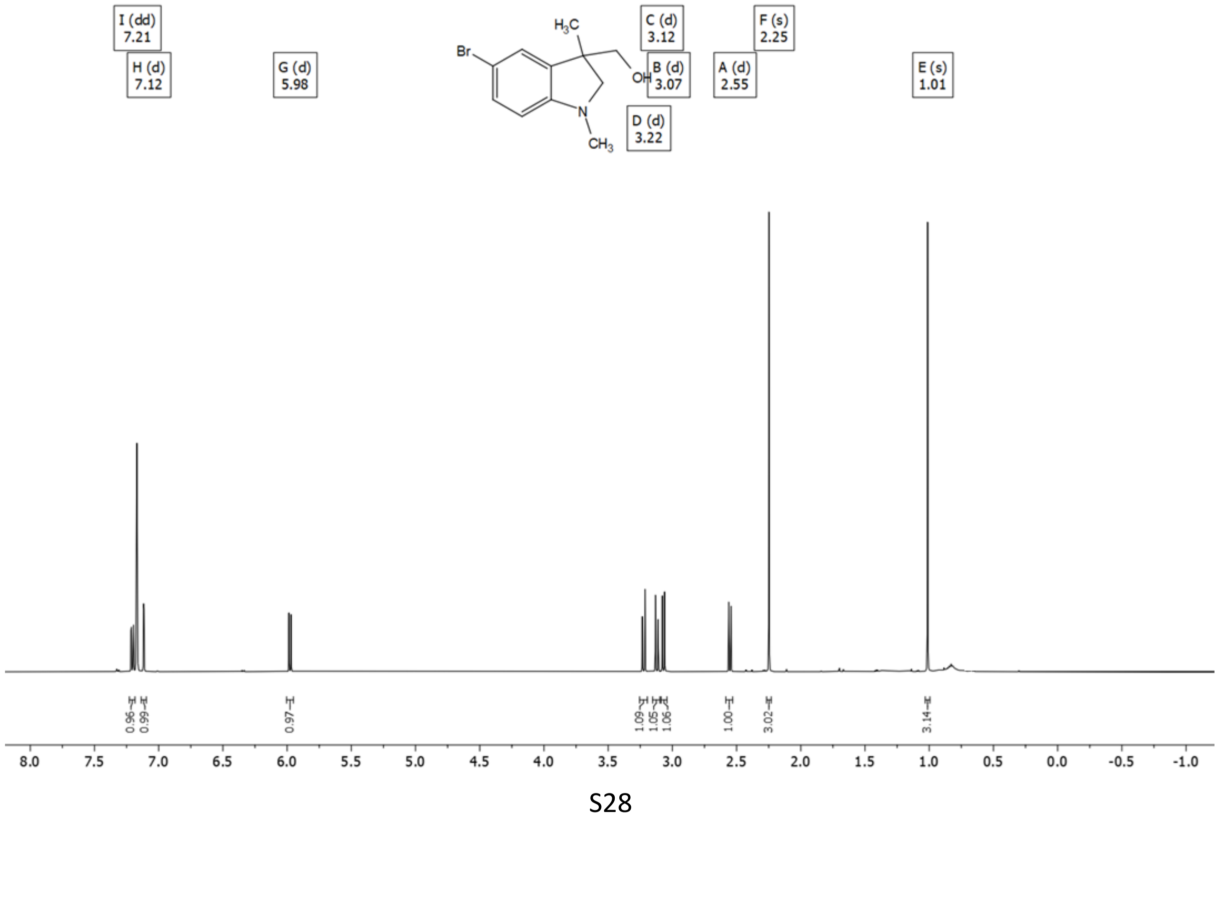

$^{13}$C NMR of **P7**

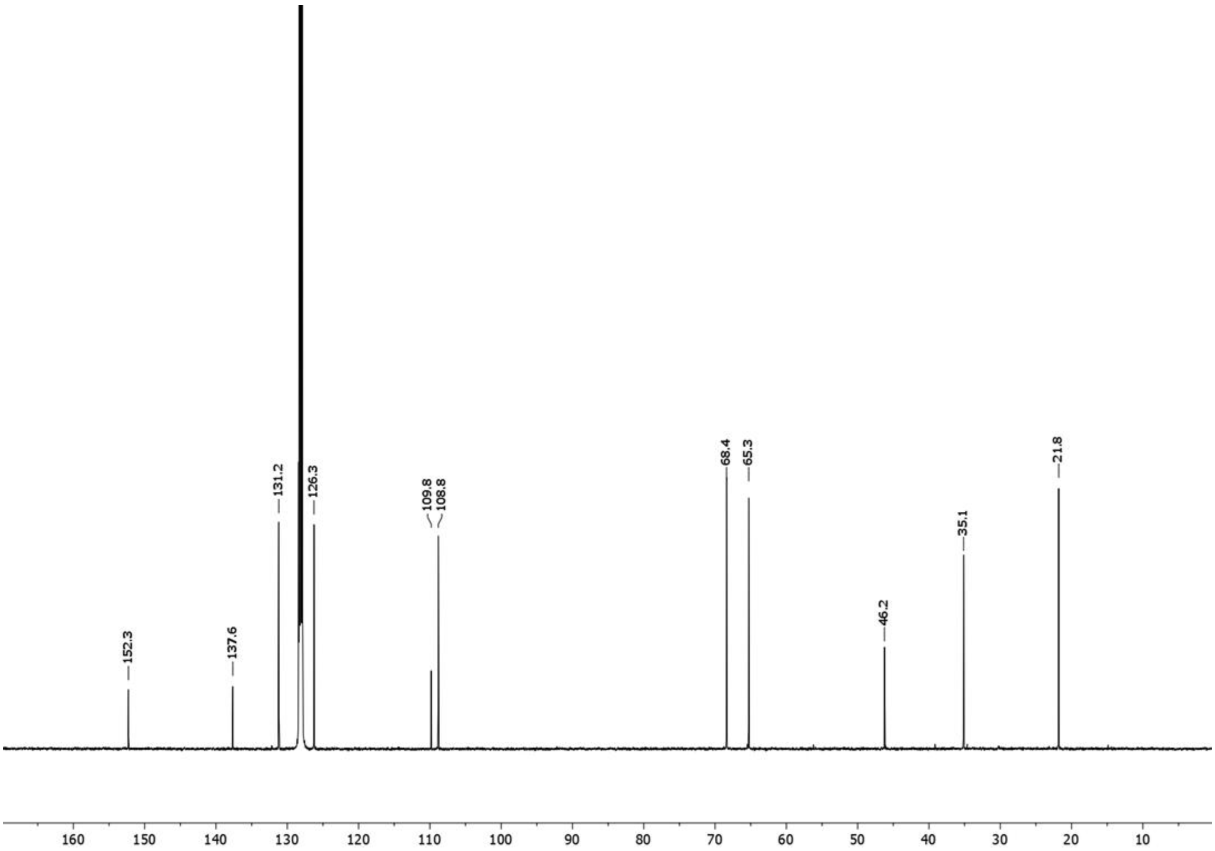

152.3
137.6
131.2
126.3
109.8
108.8
68.4
65.3
46.2
35.1
21.8
160
150
140
130
120
110
100
90
80
70
60
50
40
30
20
10

## 7. References

(1) Zhang, Z.; Richrath, R. B.; Gansäuer, A. Merging Catalysis in Single Electron Steps with Photoredox Catalysis – Efficient and Sustainable Radical Chemistry. *ACS Catal.*, **2019**, *9*, 3208-3212, https://doi.org/10.1021/acscatal.9b00787.

(2) Schmickler, N.; Gerber, S.; Hanz, L.; Grimme, S.; Qu, Z.-W; Siewert, I. Gansäuer, A. Identification of Ti(salen) Complexes for Efficient Catalysis in Single-Electron Steps by Cyclic Voltammetry. *Angew. Chem.* **2025***,* e202507673, https://doi.org/10.1002/ange.202507673.

(3) Zhang, Z.; Slak, D.; Krebs, T.; Leuschner, M.; Schmickler, N.; Kuchuk, E.; Schmidt, J.; Domenianni, L. I.; Kleine Büning, J. B.; Grimme, S.; Vöhringer, P.; Gansäuer, A. A Chiral Titanocene Complex as Regiodivergent Photoredox Catalyst: Synthetic Scope and Mechanism of Catalyst Generation. *J. Am. Chem. Soc.* **2023**, *145*, 26667-26677, https://doi.org/10.1021/jacs.3c08029.

(4) Ruos, M. E.; Garrison Kinney, R.; Ring, O. T.; Doyle, A. G. A General Photocatalytic Strategy for Nucleophilic Amination of Primary and Secondary Benzylic C-H Bonds. *J. Am. Chem. Soc.* **2023**, *145*, 18487-18496, https://doi.org/10.1021/jacs.3c04912.
